# Gene expression signatures from single‐cell transcriptomics predict Sjögren's syndrome

**DOI:** 10.1002/imo2.70106

**Published:** 2026-05-28

**Authors:** Shumin Cheng, Ling Yan, Yilin Wu, Yan Li, Ziheng Ye, Zhen Zhang, Yi Zhang, Jingyun Ma, Yuhong Liang, Zhaofan Luo, Huacheng Wang, Meili Gao, Chao Qin, Ke Zhu, Yun Leng, Kamran Ullah, Jun Liang, Haiyan Yan, Guan Yang, Yingqian Mo, Bihui Huang

**Affiliations:** ^1^ The Seventh Affiliated Hospital Sun Yat‐sen University Shenzhen China; ^2^ Shenzhen University of Advanced Technology General Hospital Shenzhen China; ^3^ Shenshan Medical Center, Sun Yat‐sen Memorial Hospital Sun Yat‐Sen University Shanwei China; ^4^ School of Pharmacy Macau University of Science and Technology Macau China; ^5^ The Second People's Hospital of Futian District Shenzhen China; ^6^ Department of Biology The University of Haripur Haripur KP Pakistan; ^7^ Department of Infectious Diseases and Public Health City University of Hong Kong Hong Kong China; ^8^ Sun Yat‐sen Memorial Hospital Sun Yat‐sen University Guangzhou China; ^9^ The People's Hospital of Guangxi Zhuang Autonomous Region Nanning China

**Keywords:** immune landscape, predictive signature, single‐cell RNA sequencing, Sjögren's syndrome

## Abstract

Sjögren's syndrome (SjS) is a systemic autoimmune disorder characterized by lymphocytic infiltration of exocrine glands, leading to dry eyes and mouth. While previous genome‐wide association studies (GWAS) and transcriptomic analyses have identified genes associated with SjS, predictive models based on single‐cell resolution are limited. In this study, single‐cell RNA sequencing (scRNA‐seq) data from peripheral blood mononuclear cells (PBMCs) of SjS patients were analyzed to map immune cell alterations linked to the disease. Compared with healthy controls, SjS patients displayed decreased proportions of naïve *CD8* ^+^ T cells and *Helios FOXP3* ^*lo*^ *CD4* ^+^ Tregs, alongside increased frequencies of *CTLA*−4^+^ *CD8* ^+^ inhibitory T cells and *TRDC* γδ T cells. Using machine learning, a predictive model for SjS diagnosis was developed based on a 12‐gene signature (SjS. Sig: *GIMAP7*, *PSMB8*, *CD27*, *CCR7*, *TAGAP*, *UQCR10*, *HCLS1*, *LCK*, *TNFAIP3*, *ISG15*, *GIMAP4*, and *HLA*‐*DRB1*), which effectively differentiated patients from healthy individuals. Key genes such as *CD27*, *PSMB8*, *HCLS1*, *LCK*, *UQCR10*, and *GIMAP4* were validated in clinical samples through flow cytometry and real‐time quantitative PCR. These findings provide insights into the immune landscape of SjS at a single‐cell resolution and propose a reliable molecular signature for diagnosis and immune monitoring.

## Highlights

- Decreased levels of Naïve *CD8* ^+^ T cells and *Helios FOXP3* ^*lo*^ *CD4* ^+^ Tregs in SjS patients.
- Increased levels of *CTLA*−4^+^ *CD8* ^+^ inhibitory T cells and *TRDC* γδ T cells in SjS patients.
- Elevated expression of *CD27*, *PSMB8*, *HCLS1*, *LCK*, *UQCR10*, and *GIMAP4* in SjS patients.
- Development and validation of a scRNA‐seq‐based predictive model for SjS diagnosis.

## Graphical abstract

A machine learning‐based 12‐gene signature (SjS.Sig) was developed from scRNA‐seq data of PBMCs from Sjögren's syndrome patients. The model was validated using bulk RNA‐seq datasets and further confirmed through flow cytometry and qPCR analysis in an independent clinical cohort, demonstrating robust diagnostic potential.

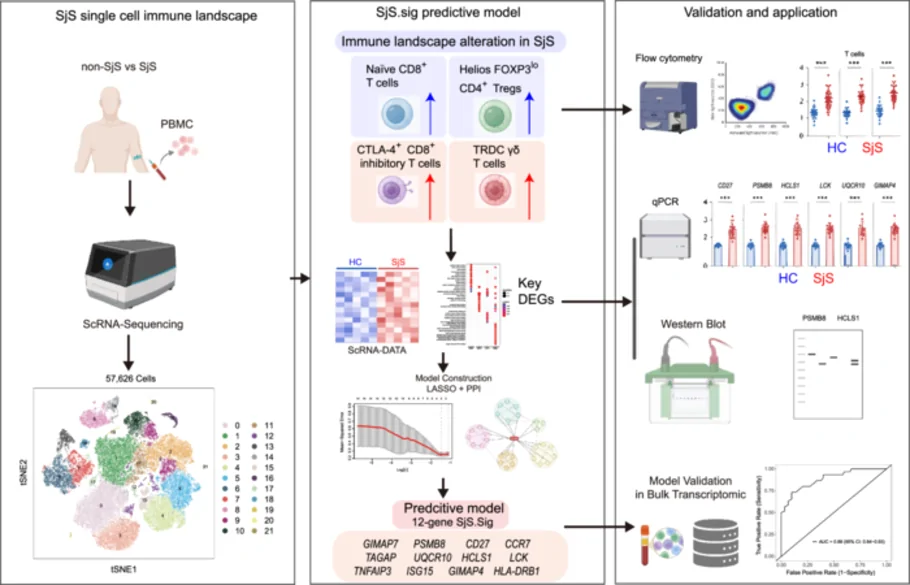

## 1 INTRODUCTION

Sjögren's syndrome (SjS) is a common autoimmune disorder predominantly affecting middle‐aged to elderly women, with a global prevalence of approximately 0.06%. The disease demonstrates a male‐to‐female ratio of 1:9, typically presenting in individuals over 50 years of age [1]. SjS is characterized by lymphocytic infiltration of the exocrine glands, particularly the salivary and lacrimal glands. However, the inflammatory process can extend beyond these glands, potentially affecting the skin, lungs, kidneys, gastrointestinal system, nervous system, and musculoskeletal system, leading to a range of symptoms including dry skin, reduced lung function, kidney damage, and peripheral neuropathy, alongside the hallmark dry mouth and eyes [2].

The pathogenesis of SjS is multifactorial, involving genetic, environmental, and immune system factors. Although the precise cause remains unclear, several genes have been implicated, including interferon regulatory factor 5 (*IRF5*), signal transducer and activator of transcription 4 (*STAT4*), interleukin‐12 subunit alpha (*IL‐12A*), and B lymphocyte kinase (*BLK*) [3, 4]. A meta‐analysis of 23 studies has identified a strong association between SjS and specific human leukocyte antigen (HLA) class II alleles (*DRB1*, *DQA1*, and *DQB1*), suggesting that antigen presentation to *CD4* ^+^ T cells and subsequent immune activation play critical roles in disease pathogenesis [5]. These associations have been further supported by genome‐wide association studies (GWAS) conducted on populations of European and Han Chinese descent [6, 7].

Currently, SjS diagnosis relies on a combination of clinical assessments (notably chronic dryness of the eyes and mouth), ocular tests (such as the Schirmer test), laboratory tests (including serological testing and minor salivary gland biopsy), and imaging studies to evaluate salivary gland function [8, 9]. However, achieving a precise differential diagnosis remains challenging due to the disease's heterogeneity, complex clinical phenotypes, and absence of specific molecular biomarkers, especially in the early stages. With the rapid advancements in single‐cell RNA sequencing (scRNA‐seq) technology and the availability of relevant datasets, identifying characteristic SjS signatures at the single‐cell level is now possible.

This study conducted an in‐depth analysis of the publicly available SjS scRNA‐seq dataset (GSE157278), focusing on immune cell molecular signatures to develop a novel predictive model for SjS. Four key immune cell subpopulations were identified as being significantly associated with SjS onset: Naïve *CD8* ^+^ T cells, *TRDC* γδ T cells, *Helios FOXP3* ^*lo*^ *CD4* ^+^ regulatory T cells (Tregs), and *CTLA*‐*4* ^*+*^ *CD8* ^+^ inhibitory T cells. These subpopulations form the biological foundation for the SjS.Sig diagnostic model, integrating their characteristic gene expression signatures (*e.g*., *PSMB8*, *CD27*, *UQCR10*) to capture immune dysregulation specific to SjS. Based on these findings, this study constructed the SjS.Sig model and validated its predictive performance using six independent bulk RNA‐seq datasets. A core set of seven genes with high diagnostic potential was identified, including two SjS‐specific differentially expressed genes (*PSMB8*, *CD27*). The model's accuracy was further validated using newly collected samples from SjS patients through flow cytometry, real‐time quantitative PCR (qPCR), and immunoblotting. This study enhances understanding of SjS pathogenesis and provides a clinically applicable diagnostic tool based on key immune cell signatures for early and accurate disease detection.

## 2 RESULTS

### 2.1 Reanalysis of scRNA‐seq data reveals cell subpopulations associated with SjS

Using the 10x Genomics platform, this study reanalyzed scRNA‐seq data from 10 participants (five SjS patients and five healthy controls [HCs]) from a previous study [10]. After performing quality control and data integration, a total of 57,626 single cells were obtained. Clustering was performed using the “clustree” R package with a resolution of 0.8, resulting in 22 initial clusters (Figure 1A and Figure S1A,B) [11]. Cell annotation was carried out by integrating automatic annotations from “SingleR” (Figure S1C), marker genes from “CellMarker” [12], and literature‐reported markers [13, 14, 15, 16, 17, 18, 19, 20, 21], leading to the identification of 10 major cell types (Figure 1B).

**Figure 1:**
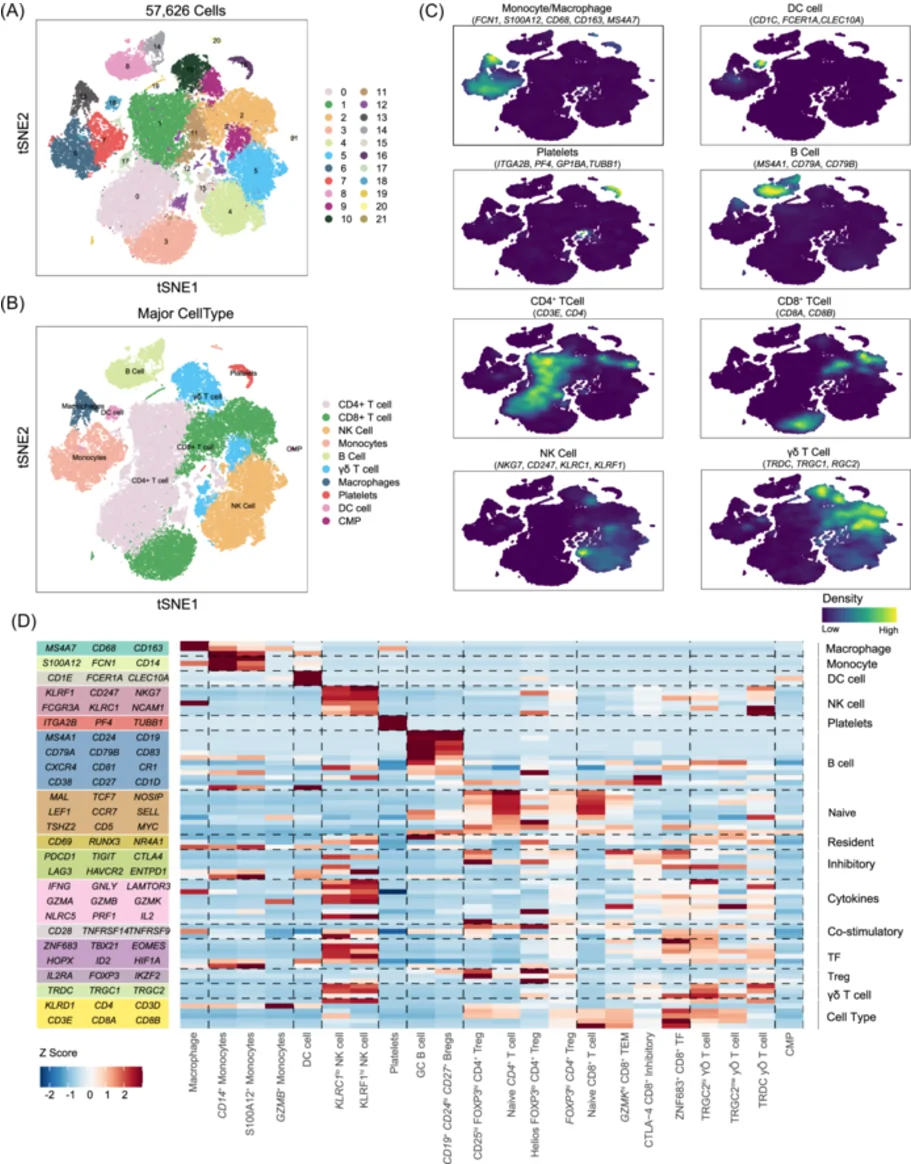
Cell clustering and annotation of GSE157278. (A) t‐SNE plot after clustering. Different colors represent the clusters obtained from 22 clusters. (B) Annotated t‐SNE maps of cell categories obtained by different techniques. (C) Nebulosa density map. The highlighted area shows a region of high expression density for multiple genes. (D) Heatmap showing the expression of selected gene sets in each cluster. *CD4* ^+^ and *CD8* ^+^ are divided into naïve, resident, inhibitory, cytokine, co‐stimulatory, transcription factor (TF), and regulatory (Treg). Rows are divided by different cell types, and columns are divided by 10 major cell types. The genes on the left side are listed in the following order from top to bottom (in the rows). t‐SNE, t‐distributed stochastic neighbor embedding.

Monocytes/macrophages were defined by high expression of *CD68*, *CD163*, *MS4A7*, *FCN1*, and *S100A12* [15, 21]. Dendritic cells (DCs) exhibited high expression of *CD1C*, *FCER1A*, and *CLEC10A* [22]. Natural killer (NK) cells were characterized by elevated levels of *NKG7*, *CD247*, *KLRC1*, and *KLRF1* [23]. Platelets displayed high expression of *ITGA2B* (*CD41*), *PF4*, *GP1BA* (*CD42b*), and *TUBB1* [24]. B cells were identified by high expression of *MS4A1* (*CD20*), *CD79A*, and *CD79B* [25]. T cells expressed *CD3D*, *CD3E*, *CD4*, *CD8A*, *CD8B*, *TRDC*, *TRGC1*, and *TRGC2* [26, 27, 28], and were further categorized into three main subgroups: CD4^+^, CD8^+^, and γδ T cells (Figure 1C). Finally, the 22 cell subpopulations were precisely annotated following the method described by Sun et al. [29] (Figure 1D).

This study analyzed the percentage of each cluster relative to the total in each participant and compared the differences between HCs and SjS patients (Figure S2A−C and Table S1). Among the 22 clusters, cluster 3 (Naïve *CD8* ^+^ T) and cluster 17 (*Helios FOXP3* ^*lo*^ *CD4* ^+^ Tregs) were significantly reduced in SjS patients compared with HCs (*p* < 0.05). In contrast, cluster 15 (*TRDC* γδ T) and cluster 19 (*CTLA*‐*4* ^*+*^ CD8^+^ inhibitory T cells) were significantly increased in SjS patients (*p* < 0.05). These four cell clusters were selected for further analysis.

### 2.2 Naïve CD8^+^ T (cluster 3) was decreased in SjS

Cluster 3 accounted for 39% of the total *CD8* ^+^ T cells, which included clusters 2, 3, 11, and 19 (Figures 1D and 2A). This cluster was characterized by high expression of *LINC02446*, *PASK*, *LEF1*, and *CCR7* (Figure 2B,C and Table S2). Differential expression analysis (“FindMarkers”) identified specific overexpression of genes such as *SNHG25*, *GIMAP5*, *GIMAP4*, *PPP1CA*, *CD27*, *GIMAP7*, *YPEL3*, *LINC01619*, *SELENOH*, and *KRT10* in this cluster (Figure 2D and Table S3). Pathway enrichment analysis based on cluster‐specific genes identified by Seurat revealed that naïve T cells were enriched in pathways such as structural constituent of ribosome, rRNA binding, translation regulation, mRNA 5’‐UTR binding, and ubiquitin‐protein transferase regulator activity (Figure 2E and Table S4). These findings align with the relatively high expression of ribosomal proteins (*RPS3A*, *RPS5*, *RPS18*) and eukaryotic translation elongation factors (*EEF1B2*, *EEF1G*) in Naïve *CD8* ^+^ T cells.

**Figure 2:**
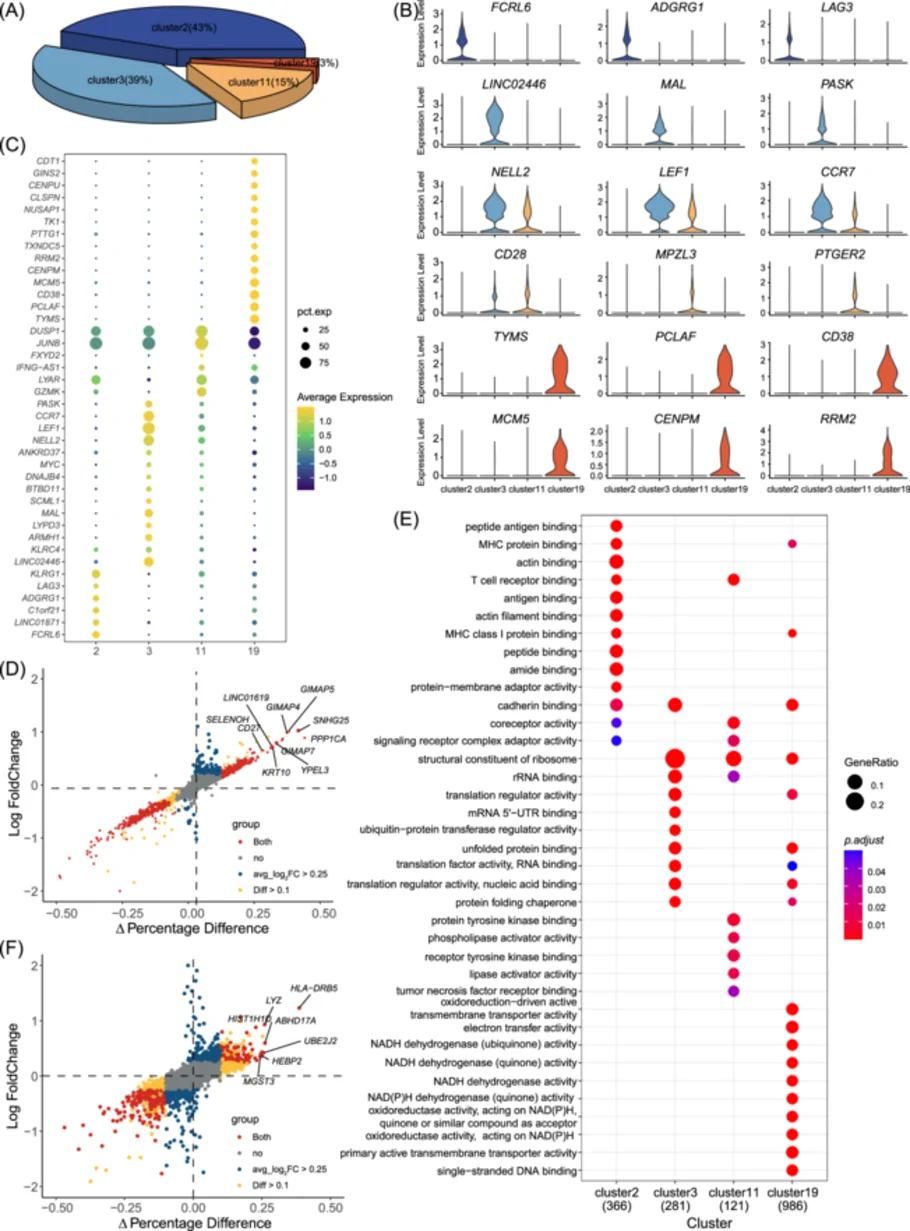
Correlation analysis of *CD8* ^+^ T cell subsets in GSE157278. (A) 3D pie chart of the proportion of each subset of *CD8* ^+^ T cells. The 10‐marked genes represent the top 10 most differentially expressed genes in SjS patients. (B) Violin plot of log‐transformed expression of specifically expressed genes, showing statistically significant upregulation in each *CD8* ^+^ T cell subset. (C) Bubble diagram of highly expressed genes in each subgroup of *CD8* ^+^ T cells [*ZNF683* ^+^ *CD8* ^+^ TF (cluster 2), Naïve *CD8* ^+^ T cell (cluster 3), *GZMK* ^high^ *CD8* ^+^ TEM (cluster 11), and *CTLA‐4*⁺ *CD8* ^+^ Inhibitory (cluster 19)], the size of the point represents the percentage expression, and the color of the point represents the size of the expression, ranging in color from purple to yellow. (D) Cross‐volcano plots of gene expression in Naïve *CD8* ^+^ T cells (cluster 3) subpopulations. (E) Gene Ontology (GO) enrichment analysis map in each subpopulation of *CD8* ^+^ T cells. (F) Cross‐volcano plots of gene expression in *CTLA*‐*4⁺ CD8* ^+^ Inhibitory (cluster 19) subpopulations.

### 2.3 *CTLA‐4* ^+^ CD8^+^ inhibitory (cluster 19) was increased in SjS

Cluster 19 accounted for 3% of the total CD8^+^ T cells (Figure 2A). This cluster exhibited overexpression of genes such as *TYMS*, *PCLAF*, *CD38*, *MCM5*, *CENPM*, and *RRM2* (Figure 2B,C and Table S5). Compared with other *CD8* ^+^ T‐cell clusters, this cluster demonstrated significantly higher expression of immune checkpoint inhibitors, including *CTLA‐4*, *TIGIT*, and *PDCD1* (Figure 1D), and was thus designated *CTLA‐4* ^+^ *CD8* ^+^ inhibitory T cells [30]. Differential expression analysis using the “FindMarkers” function revealed high expression of *HLA*‐*DRB5*, *ABHD17A*, *UBE2J2*, *LYZ*, *TAF10*, *HEBP2*, *EMC6*, *TRG*‐*AS1*, *HIST1H1D*, and *MGST3* (Figure 2F and Table S4). Pathway enrichment analysis indicated that *CTLA*‐*4* ^*+*^ CD8^+^ inhibitory T cells (cluster 19) were significantly enriched in pathways related to energy metabolism, including oxidoreduction‐driven active transmembrane transporter activity, electron transfer activity, NADH dehydrogenase activity, primary active transmembrane transporter activity, and single‐stranded DNA binding (Figure 2E).

### 2.4 *TRDC* γδ T cell (cluster 15) was increased in SjS

Among the γδ T cell populations (clusters 9, 10, and 15), only cluster 15 exhibited a significant percentage difference between groups (Figure 1D and Figure S2C; *p* < 0.05). This cluster represented 18% of γδ T cells (Figure S3A). In contrast to other γδ T cell clusters, *TRDC* γδ T cells displayed high expression of *XCL1*, *KLRF1*, *KLRC2*, and *TRDC* (Figure S3B,C, and Table S6). Differential expression analysis revealed that the *TRDC* γδ T cells from SjS patients exhibited characteristic overexpression of *HLA*‐*DRB5*, *PPP1CA*, *MYOM2*, *S100A11*, *GIMAP7*, *YPEL3*, and *GIMAP4* (Figure S3D and Table S7). Pathway enrichment analysis demonstrated that *TRDC* γδ T cells were enriched for gene ontology (GO) terms such as phosphotyrosine residue binding, nonmembrane spanning protein tyrosine kinase activity, and retinoic acid receptor binding (Figure S3E and Table S8).

### 2.5 Helios FOXP3^lo^ CD4^+^ Treg (cluster 17) was decreased in SjS

Among the *CD4* ^+^ T cell populations (clusters 0, 1, 12, and 17; Figure 1D), only cluster 17 exhibited a significant percentage difference between groups (Figure S2C; *p* < 0.05). This cluster accounted for 4% of *CD4* ^+^ T cells (Figure S4A) and was characterized by high expression of *MED13L*, *ZSWIM6*, *IL2RA*, and *IKZF2*, and was thus designated *Helios FOXP3* ^*lo*^ *CD4* ^+^ Treg (Figure S4B,C and Table S9). Differential expression analysis identified characteristic overexpression of *LINC01619*, *PPBP*, *IL2RG*, and *SNHG25* in this subgroup (Figure S4D and Table S10). Pathway enrichment analysis indicated that the *Helios FOXP3* ^*lo*^ *CD4* ^+^ Treg subpopulation was enriched for terms related to protein serine kinase activity, GTPase regulator activity, nucleoside‐triphosphatase regulator activity, transcription coregulator activity, and ubiquitin‐like protein transferase activity (Table S11).

### 2.6 Validation of four featured clusters

The overexpressed genes and pathway‐enriched genes for the four previously identified clusters were compiled, and gene set scoring (GSS) and enrichment methods were applied for single‐cell gene scoring, confirming the specificity of each cluster (Figure 3A,B and Figure S5A,B). Genes highly expressed in SjS patients relative to HCs were then selected. These candidate genes underwent training using Least Absolute Shrinkage and Selection Operator (LASSO) regression across six publicly available gene expression datasets (Table 1 and Figures S6−S9). The results indicated that gene set variation analysis (GSVA) scores for each SjS patient cluster were significantly higher than those for the HCs (Figure 3C,D and Figure S5C,D). Detailed analyses of three SjS‐specific subsets, including heatmaps of highly expressed genes in *CD8* ^+^ T cells, γδ T cells, and *CD4* ^+^ T cells, as well as network correlation diagrams of GO term enrichment for each subset, are provided in Figure S10. Ultimately, cellular signatures for cluster 3 (20 genes), cluster 19 (21 genes), cluster 15 (23 genes), and cluster 17 (19 genes) were established (Table S12). Fluorescence‐activated cell sorting (FACS) analysis of peripheral blood mononuclear cells (PBMCs) from 20 participants (9 HCs and 11 SjS patients; Table S13) confirmed these findings (Figure 4A–D). Compared with controls, cluster 3 (Naïve *CD8* ^+^ T cells) and cluster 17 (*Helios FOXP3* ^*lo*^ *CD4* ^+^ Treg) were significantly reduced, while cluster 15 (*TRDC* γδ T cells) and cluster 19 (*CTLA*‐*4* ^*+*^ *CD8* ^+^ Inhibitory) were significantly elevated in SjS patients (Figure 4E–H; *p* < 0.05). Subsequent transcriptional validation of these signature genes is presented in Figure 4I–N, following the construction of the prediction model.

**Figure 3:**
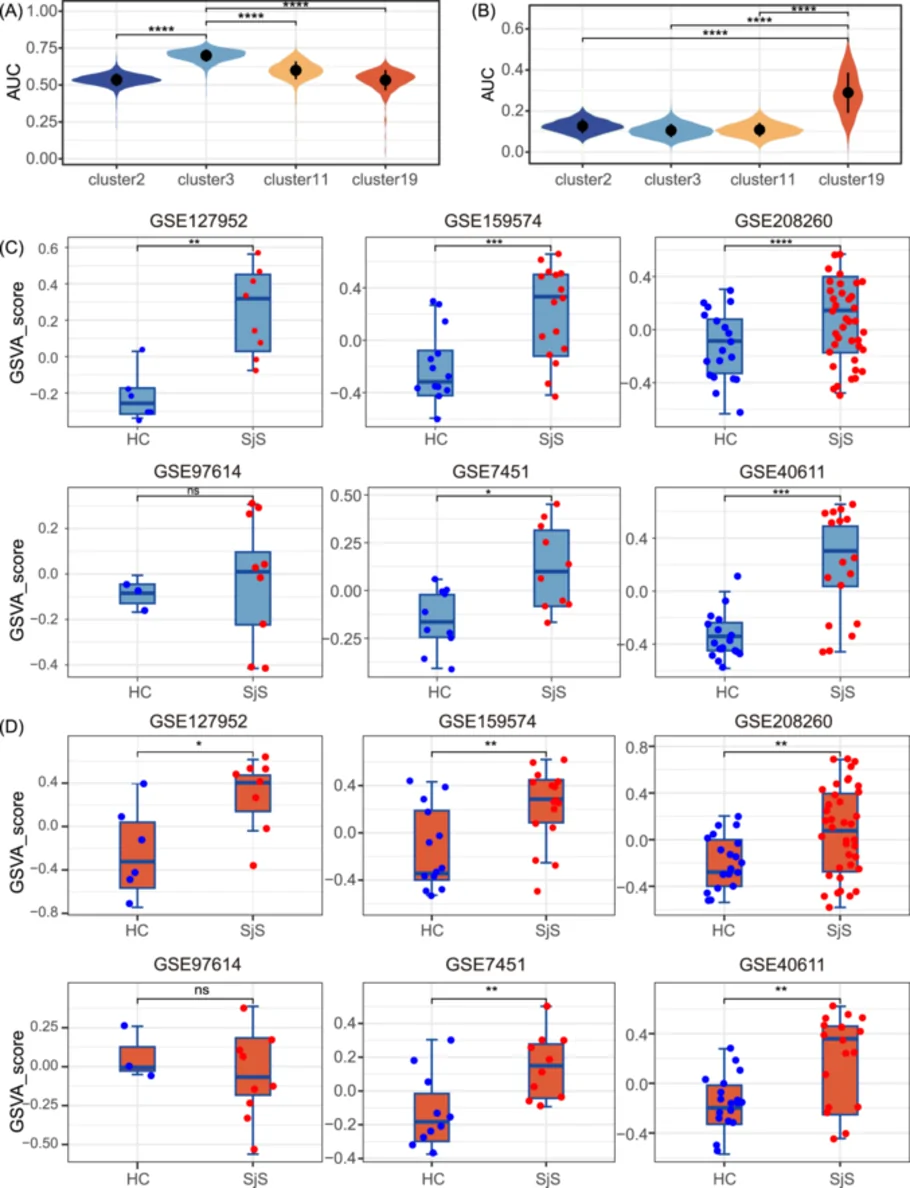
Gene expression signatures that can characterize Naïve *CD8* ^+^ T cells and *CTLA‐4^+^ CD8* ^+^ Inhibitory subpopulations. (A) AUCell score of the characteristic expression gene set in Naïve *CD8*⁺ T cells. The violin plot shows that this gene set is more active in Naïve *CD8*⁺ T cells than in other *CD8*⁺ T cell subsets. (B) AUCell scores for the signature expressed gene sets of the *CTLA*‐*4⁺ CD8* ^+^ Inhibitory subpopulation. (C) After LASSO regression analysis, the GSVA analysis of the characteristic expression gene sets (*n* = 20) related to SjS in the Naïve *CD8* ^+^ T cell subpopulation in the six gene expression omnibus (GEO) test datasets, the box plot shows that the Naïve *CD8* ^+^ T‐cell genome of SjS Patients had significantly higher GSVA scores than those of healthy controls (HC). (D) GSVA analysis of the characteristic expression gene sets (*n* = 21) associated with SjS in the *CTLA*‐*4⁺* CD8^+^ Inhibitory subpopulation in the six GEO test datasets after lasso regression analysis. Significant differences are indicated by *p < 0.05, **p < 0.01, ***p < 0.001, ****p < 0.0001, ns: not significant, as shown in the corresponding panels.

**Table 1:** Selected gene expression omnibus (GEO) datasets for bioinformatics analysis.

GEO datasets	RNA library	Platform	samples		Data category	Application
			Healthy	SjS		
GSE157278	Single‐cell RNA sequencing	GPL24676	5	5	Expression profiling by high‐throughput sequencing	Analysis
GSE208260	Bulk RNA sequencing	GPL16791	20	39	Expression profiling by high‐throughput sequencing	Training
GSE159574	Bulk RNA sequencing	GPL20301	13	16	Expression profiling by high‐throughput sequencing	Training
GSE127952	array	GPL20995	6	8	Expression profiling by array	Training
GSE97614	array	GPL6244	3	9	Expression profiling by array	Training
GSE40611	array	GPL570	18	17	Expression profiling by array	Training
GSE7451	array	GPL570	10	10	Expression profiling by array	Training
GSE84844	array	GPL570	30	30	Expression profiling by array	Predict
GSE143153	array	GPL13607	15	17	Expression profiling by array	Predict

Abbreviation: SjS, Sjögren's syndrome.

**Figure 4:**
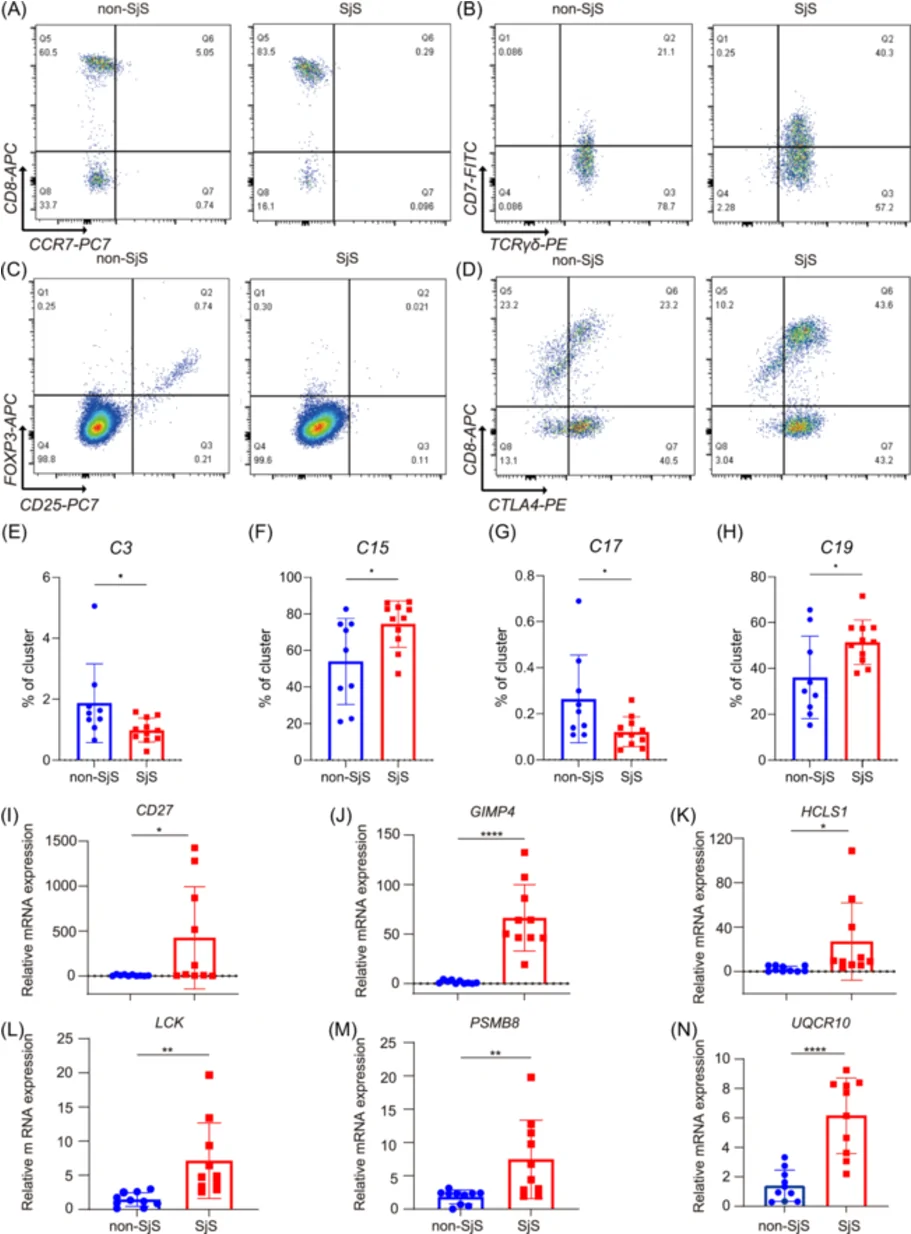
Validation of featured clusters and differentially expressed genes in human peripheral blood mononuclear cells. (A) Flow cytometry dot plot showing the distribution of *CD8 versus CCR7* expression in CD3^+^ T cells from non‐ Sjögren's syndrome (SjS) (left) and SjS (right) samples. (B) Flow cytometry dot plot showing the distribution of *TCRγδ versus CD7* expression in CD3^+^ T cells from non‐SjS (left) and SjS (right) samples. (C) Flow cytometry dot plot showing the distribution of *FOXP3 versus CD25* expression in CD3^+^ T cells from non‐SjS (left) and SjS (right) samples. (D) Flow cytometry dot plot showing the distribution of *CD8 versus CTLA4* expression in CD3^+^ T cells from non‐SjS (left) and SjS (right) samples. (E−H) Bar plot showing the percentage of cluster 3, cluster 15, cluster 17, cluster 19 in non‐SjS and SjS samples. (I−N) The mRNA expression levels of *CD27, GIMAP4*, *HCLS1*, *LCK*, *PSMB8*, and *UQCR10* were analyzed by quantitative PCR (qPCR). These markers were selected based on their differential expression across the different cell subsets. Significant differences are indicated by *p < 0.05, **p < 0.01, ***p < 0.001, ****p < 0.0001, as shown in the corresponding panels.

### 2.7 Construction and validation of a gene prediction model

To refine the prediction model, characteristic genes from the four highlighted clusters were consolidated, with duplicates removed (Table S12). A protein–protein interaction (PPI) network was constructed using search tool for the retrieval of interacting genes/proteins (STRING), comprising 14,542 nodes and 61,820 edges. Key subnetworks were identified using the Molecular Complex Detection (MCODE) algorithm, resulting in two modules: one with 106 nodes and 554 edges, and another with 154 nodes and 499 edges (score > 5; Figure 5A,B). Hub genes (degree > 20) were identified through CytoHubba, and their intersection with the characteristic genes from the four clusters led to the development of a 12‐gene signature (*GIMAP7*, *PSMB8*, *CD27*, *CCR7*, *TAGAP*, *UQCR10*, *HCLS1*, *LCK*, *TNFAIP3*, *ISG15*, *GIMAP4*, and *HLA‐DRB1*), hereafter referred to as SjS.Sig, reflecting their potential regulatory roles in SjS (Figure 5B).

**Figure 5:**
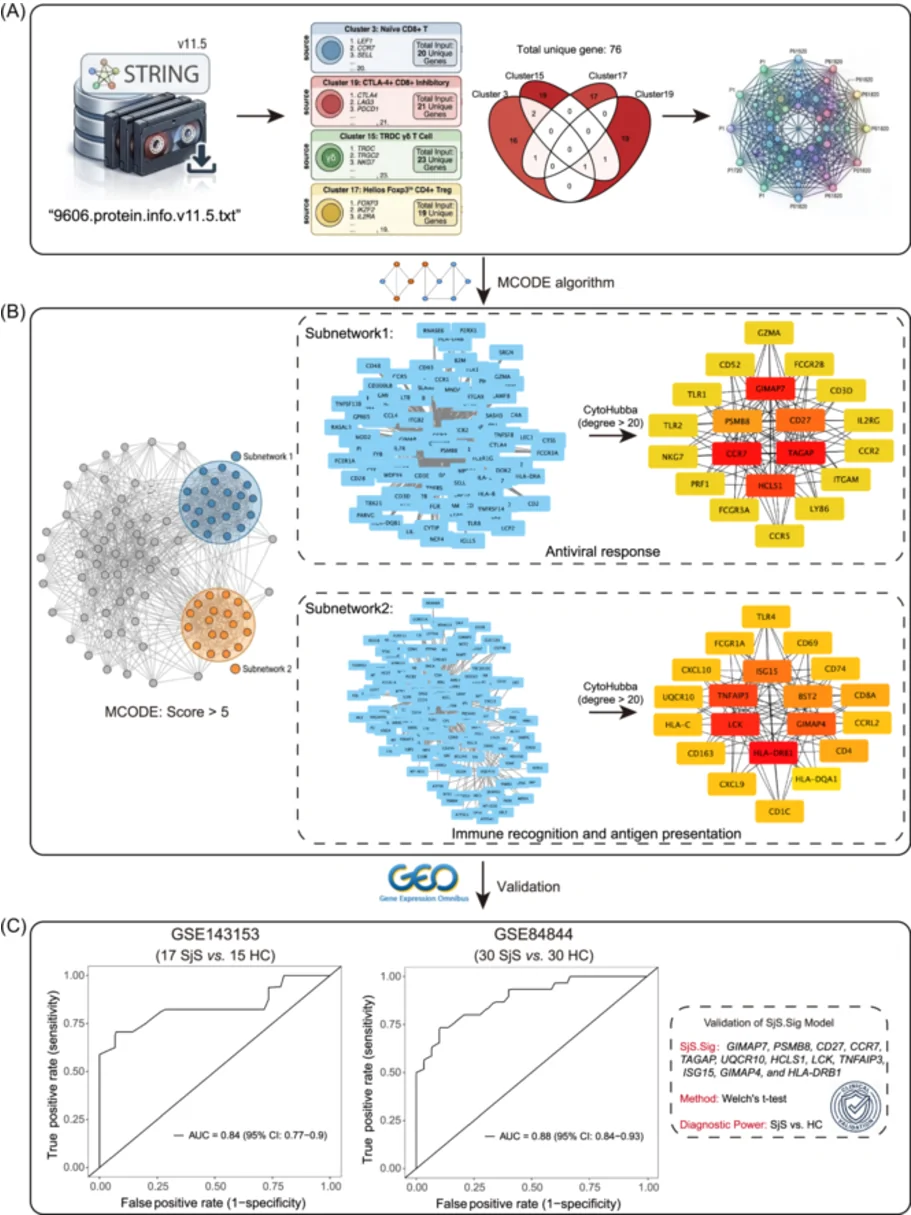
Establishment of protein–protein interaction (PPI) network and determination of SjS.Sig. (A) The total PPI network constructed according to STRING contains 14,542 nodes and 61,820 edges. (B) Molecular Complex Detection (MCODE) subnetwork analysis and hub gene identification. Two significant gene clusters (score > 5) were extracted using MCODE: subnetwork1 (106 nodes, 554 edges, up) and subnetwork2 (154 nodes, 499 edges, down). The central expanded nodes represent the top 10 hub genes identified by the CytoHubba Degree algorithm, highlighting the core regulatory components within each functional cluster. (C) The predictive performance of SjS.Sig when validating GSE143153 and GSE84844.

To verify the predictive performance of SjS.Sig, it was evaluated in two independent gene expression datasets (GSE84844 and GSE143153), neither of which was used in the model development. For the GSE84844 dataset (*n* = 60, HCs *vs*. SjS: 30 *vs*. 30), SjS.Sig successfully distinguished HCs from SjS patients with an area under the curve (AUC) of 0.88 (95% confidence interval [CI], 84%–93%; Figure 5C), sensitivity of 89% (95% CI, 80%–94%), and specificity of 87% (95% CI, 72%–95%). Similarly, for the GSE143153 dataset (*n* = 32, HCs *vs*. SjS: 15 *vs*. 17), SjS.Sig achieved an AUC of 0.84 (95% CI, 77%–90%; Figure 5C), sensitivity of 85% (95% CI, 77%–92%), and specificity of 82% (95% CI, 65%–92%). These results demonstrate that SjS.Sig is an effective biomarker for accurately predicting SjS patients.

Calibration plots and Brier scores confirmed both strong discrimination and reliable calibration (Figure S11A,B). These additional analyses reinforce that SjS.Sig not only offers excellent discriminatory power but also shows reliable calibration, enhancing its potential as a robust biomarker for SjS prediction. Furthermore, the performance of SjS.Sig in rheumatoid arthritis (RA) was assessed through receiver operating characteristic (ROC) analysis, calibration plots, and Brier scores (Figure S11C–F). The results indicated poor performance in RA, further validating the specificity of SjS.Sig for SjS.

Six genes (*PSMB8*, *CD27*, *UQCR10*, *HCLS1*, *LCK*, and *GIMAP4*) were significantly upregulated in SjS patients in scRNA‐seq data (Figure S12). To validate their expression and predictive value, new blood samples were collected from 20 participants (10 non‐SjS and 10 SjS patients; Table S14) to compare the transcriptional levels of these genes between the two groups using real‐time qPCR. The results showed that all six genes were significantly upregulated in the SjS group compared with controls (*p* < 0.05) (Figure 4I–N). Additionally, PBMCs from another eight participants (four non‐SjS and four SjS patients) were collected, and the protein expression levels of *PSMB8*, *HCLS1*, and *GIMAP4* were compared using Western blot analysis (Figure S13), confirming the validity of the predictive model.

To further validate the robustness of the identified gene clusters, Random Forest (RF) analysis was performed on four cell subpopulations across six Gene Expression Omnibus (GEO) datasets (Figures S14 and S15). For each dataset, genes were ranked based on their importance scores, derived from the mean decrease in accuracy (MDA) metric. Genes exceeding the predefined threshold were selected as RF‐identified features. An intersection analysis was then conducted to identify genes consistently selected across various datasets and cell populations. This integrative analysis resulted in a robust candidate gene set consisting of 21 genes.

To assess the generalizability of the RF‐derived gene clusters, external validation was performed using the independent GSE143153 and GSE84844 datasets. In the GSE143153 dataset, the identified gene cluster effectively distinguished HCs from SjS patients, achieving an AUC of 0.81 (95% CI, 0.75%–0.89%; Figure S16A). Similarly, in the GSE84844 dataset, the gene cluster demonstrated strong predictive performance with an AUC of 0.88 (95% CI, 0.85%–0.92%; Figure S16B).

Although the results from the RF analysis were slightly less optimal than those from the “LASSO + PPI” approach, the gene set identified by RF was significantly larger. This suggests that while RF may provide a broader range of potential biomarkers, the larger gene set may limit its practical applicability in clinical settings due to challenges in clinical validation and implementation.

### 2.8 Cellular crosstalk and immune dysregulation in SjS

Compared with HCs, the overall cell–cell communication strength was significantly increased in SjS (Figure 6A,B). Notably, there were distinct communication changes between specific immune cell subsets. Interactions between Naïve *CD8* ^+^ T cells and other cell populations were significantly reduced, both in terms of incoming and outgoing interaction strength (Figure 6C,D), suggesting that the immune activity of this subset may be suppressed. Intriguingly, while the overall communication of Naïve *CD8* ^+^ T cells was diminished, the activity of specific pathways diverged. We observed a decrease in major histocompatibility complex (MHC)‐I signaling, which is consistent with the dampened outgoing/incoming interaction strength of these cells. Notably, the MHC‐II pathway did not show a parallel reduction, indicating a selective alteration in communication pathways rather than a blanket suppression (Figure 6A,E,F). This dissociation might suggest a shift in the functional state or interacting partners of Naïve *CD8* ^+^ T cells in SjS, possibly related to aberrant immune regulation.

**Figure 6:**
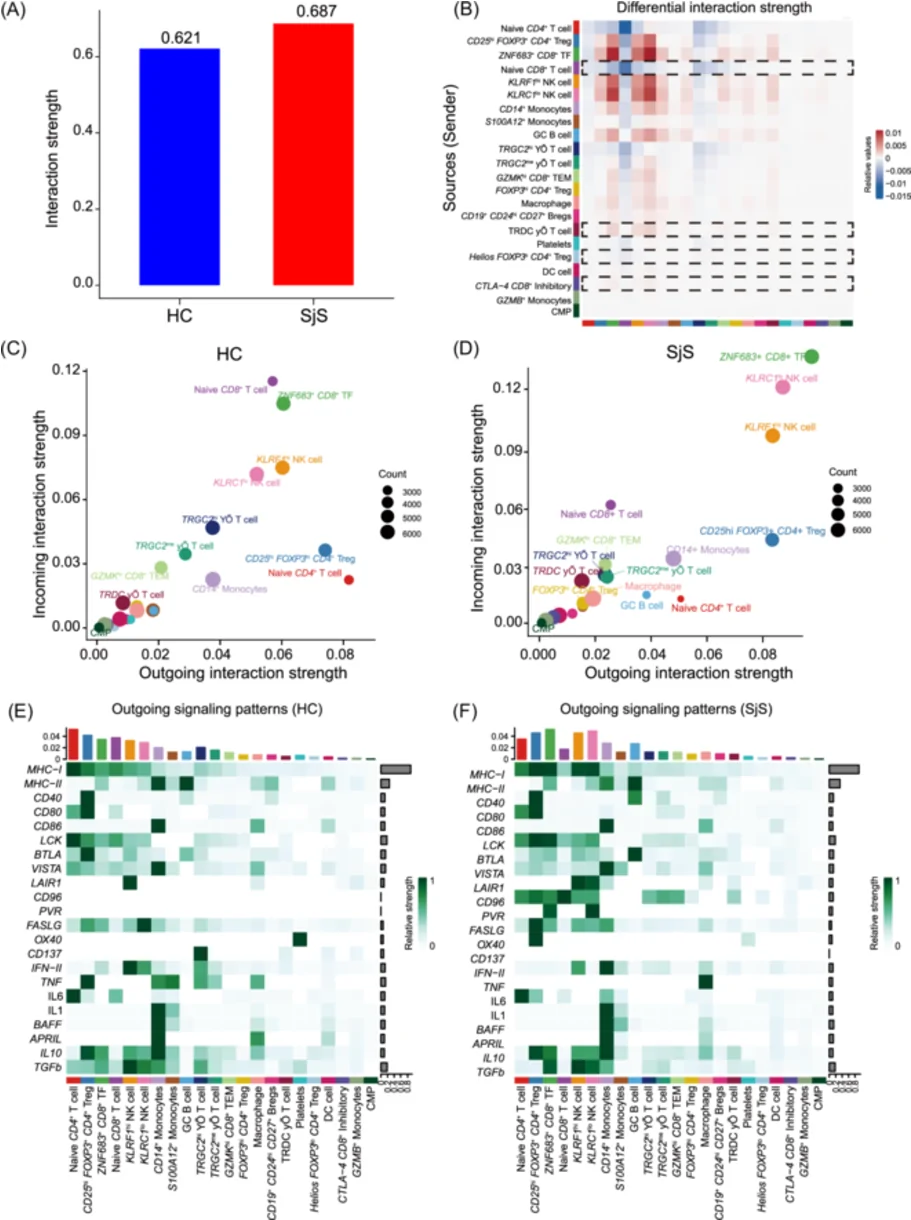
Intercellular communication and signaling patterns in healthy control (HC) and Sjögren's syndrome (SjS). (A) Bar plots showing the overall intercellular interaction strength in HC and SjS samples. (B) Heatmap of differential interaction strength (SjS *vs*. HC). The vertical axis represents the source cells (Sender), and the horizontal axis represents the target cells (Receiver). Red indicates stronger interactions in SjS, while blue represents weaker interactions. The dashed lines highlight the four specific subsets of interest. (C and D) Scatter plots of outgoing versus incoming interaction strength in HC and SjS. The x‐axis represents the outgoing interaction strength, and the y‐axis represents the incoming interaction strength. (E and F) Heatmaps of outgoing signaling patterns for major immune signaling pathways in HC and SjS. The color in the heatmap represents the relative signaling strength. The bar plot on the right indicates the total outgoing signal strength per sender cell, and the bar plot on the top shows the overall signaling strength of each pathway.

In contrast, interactions of *TRDC* γδ T cells and *CTLA*‐*4* ^*+*^ *CD8* ^+^ inhibitory T cells with other cell populations were significantly increased (Figure 6B), suggesting these subsets may be in a more activated immune state in SjS, potentially exhibiting enhanced immune‐suppressive functions. This increased cell–cell communication may be closely related to the breakdown of immune tolerance, chronic inflammation, and tissue damage in SjS.

These findings highlight the altered immune function of Naïve *CD8* ^+^ T cells in SjS, as well as the critical roles of *TRDC* γδ T cells and *CTLA*‐*4* ^*+*^ *CD8* ^+^ inhibitory T cells in immune regulation. Overall, the immune environment in SjS patients is characterized by significant reshaping of cell–cell communication patterns, which may provide valuable insights into the immune mechanisms underlying the disease.

## 3 DISCUSSION

Dysregulation of immune cells, leading to autoantibody production and subsequent tissue damage, is a key mechanism in the pathogenesis of SjS [31]. However, genetic analyses of SjS have predominantly relied on meta‐analyses, GWAS, and bulk transcriptomics, often overlooking the contributions of specific immune cell types. Moreover, existing predictive models typically utilize mixed samples, failing to capture the distinct molecular signatures of individual immune cell populations. To address this gap, this study developed a predictive model for SjS diagnosis at single‐cell resolution.

By reanalyzing publicly available scRNA‐seq data from SjS patients, this study identified four key immune cell subsets associated with disease risk: a reduction in naïve *CD8* ^+^ T cells and *Helios FOXP3* ^*lo*^ *CD4* ^+^ Tregs, along with an increase in *TRDC* γδ T cells and *CTLA*‐*4* ^*+*^ *CD8* ^+^ inhibitory T cells. These dynamic changes reflect a profound imbalance in immune homeostasis in SjS. Specifically, the reduction in *Helios FOXP3* ^*lo*^ *CD4* ^+^ Treg cells undermines immune tolerance, while the expansion of *TRDC* γδ T cells acts as an “inflammation amplifier,” exacerbating local tissue damage by secreting proinflammatory cytokines. In response to this imbalance, the increase in *CTLA*‐*4* ^*+*^ *CD8* ^+^ suppressor T cells may represent a compensatory mechanism; however, chronic inflammation often leads to the exhaustion or ineffectiveness of this suppressive function. Ultimately, the exhaustion of naïve *CD8* ^+^ T cells reflects a gradual loss of functional resilience and diversity within the immune system, driven by persistent and ineffective self‐attack. Thus, the progression of SjS is attributed not only to the overactivation of effector immunity but also to the dysfunction of regulatory functions and the depletion of immune resources.

Leveraging these findings, this study constructed SjS.Sig, a novel machine‐learning‐based diagnostic model incorporating characteristic genes from the four identified cell clusters. This biologically informed model includes 12 genes: *GIMAP7*, *PSMB8*, *CD27*, *CCR7*, *TAGAP*, *UQCR10*, *HCLS1*, *LCK*, *TNFAIP3*, *ISG15*, *GIMAP4*, and *HLA‐DRB1*. Each has biological plausibility in the context of SjS pathogenesis. The *GIMAP* gene family (*GIMAP4* and *GIMAP7*) encodes GTPases essential for lymphocyte survival and homeostasis, and their dysregulation has been implicated in autoimmune susceptibility [32]. *PSMB8*, a component of the immunoproteasome, is critically involved in MHC class I antigen processing and has been associated with type I interferon‐driven autoimmunity [33]. *CD27*, a tumor necrosis factor receptor superfamily member, regulates B‐ and T‐cell activation and differentiation; its altered expression in SjS patients may reflect dysregulated lymphocyte co‐stimulation [34]. *LCK*, a Src‐family kinase indispensable for T‐cell receptor (TCR) signal transduction, and *HCLS1*, a hematopoietic cell‐specific adaptor protein involved in antigen receptor signaling, collectively implicate dysregulated proximal TCR signaling in SjS pathophysiology. *UQCR10*, a subunit of mitochondrial respiratory chain complex III, reflects the metabolic reprogramming evident in CTLA‐4⁺ CD8⁺ inhibitory T cells, consistent with the energy metabolism pathway enrichment observed in this subpopulation. *TNFAIP3* (A20) is a ubiquitin‐editing enzyme with critical roles in terminating NF‐κB signaling and maintaining immune homeostasis; loss‐of‐function variants in *TNFAIP3* have been associated with multiple autoimmune disorders, including SjS [35]. *ISG15*, an interferon‐stimulated gene, reflects the prominent type I interferon signature that is a hallmark of SjS pathogenesis [36]. *CCR7* and *HLA‐DRB1* further corroborate the involvement of T‐cell trafficking and antigen presentation in disease mechanisms, consistent with established GWAS findings [5, 6, 7]. In addition to the characteristic genes of the four cell subpopulations, the model integrates six differentially expressed genes in SjS: *CD27*, *PSMB8*, *HCLS1*, *LCK*, *UQCR10*, and *GIMAP4*.

Notably, our study differs from the original scRNA‐seq publication due to variations in dimensionality reduction parameters and clustering nomenclature. The markers used for clustering *CD4* ^+^ T cell or *CD8* ^+^ T cell subpopulations in this study were compared with those in the original article (Figure S17 and Table S15). Notably, *CD4* ^+^ CTLs (characterized by high expression of *GZMH*, *GZMA*, and *GZMB*) and CD4^+^ *TRAV13*‐*2* ^+^ T cells (characterized by high expression of *TRAV13*‐*2*) in the original study were dispersed into *CD25* ^+^ *FOXP3* ^*hi*^ *CD4* ^+^ Tregs (cluster 1) and *FOXP3* ^*hi*^ *CD4* ^+^ Tregs (cluster 12) in this study. Similarly, Th17 cells (characterized by high expression of *RORA*, *IL6ST*, and *IL17RA*) were dispersed into Naïve CD4^+^ T cells (cluster 0) and *Helios FOXP3* ^*lo*^ *CD4* ^+^ Tregs (cluster 17), with a significant decrease in *Helios FOXP3* ^*lo*^ *CD4* ^+^ Tregs observed in SjS. Additionally, due to more detailed clustering of *CD8* ^+^ T cells, naïve *CD8* ^+^ T cells and *CTLA*‐*4* ^*+*^ *CD8* ^+^ inhibitory T cells exhibited characteristic immune features of SjS, despite neither *CD8* ^+^ T‐cell subpopulation reaching statistical significance in the original study. The new findings in this study were validated by a substantial amount of bulk RNA‐seq data and clinical fresh samples.

While this study offers novel insights and a promising diagnostic tool for SjS, several limitations warrant consideration. First, the discovery‐phase scRNA‐seq cohort (GSE157278) consisted of a relatively small sample size (five SjS patients and five HCs), which is a common limitation in initial exploratory single‐cell studies. To address this, a comprehensive validation strategy was employed. The identified cellular alterations were validated through flow cytometry in an independent clinical cohort, and the molecular signature (SjS.Sig) was rigorously tested across multiple larger bulk transcriptomic datasets and by qPCR in fresh samples. Second, while genes in the SjS.Sig are implicated in immune regulation and metabolism; the precise functional roles of certain genes (*e.g*., *HCLS1*, *UQCR10*) in SjS pathogenesis remain unclear and require further mechanistic investigation. Third, the model is primarily based on peripheral blood signatures; future studies should examine its correlation with tissue‐specific immune pathology in salivary glands and assess its utility in monitoring disease activity or treatment response.

In summary, this study presents the first scRNA‐seq‐based predictive model for SjS diagnosis, integrating single‐cell molecular signatures with machine‐learning approaches. Given the accessibility of peripheral blood testing, SjS.Sig offers a practical and robust tool for clinical applications, providing both diagnostic accuracy and valuable insights into disease pathogenesis.

## 4 CONCLUSION

In conclusion, through the re‐analysis of scRNA‐seq data from PBMCs of SjS patients, Naïve *CD8* ^+^ T cells, *Helios FOXP3* ^*lo*^ *CD4* ^+^ Treg cells, *TRDC* γδ T cells, and *CTLA*‐*4* ^*+*^ *CD8* ^+^ inhibitory T cells were identified as key differentiators between SjS patients and HCs. Additionally, this study introduces a novel prediction model (SjS.Sig) utilizing machine learning methods at the single‐cell level and validates it through a clinical cohort, providing a new tool for clinical diagnosis and prognosis of SjS.

## 5 METHODS

### 5.1 Study design

Single‐cell RNA‐sequencing (scRNA‐seq) data of PBMCs from SjS patients and HCs from the original study (accession number GEO: GSE157278) were downloaded and reanalyzed [10]. For training, six transcriptome‐level gene expression datasets (accession numbers: GSE208260, GSE159574, GSE127952, GSE97614, GSE40611, and GSE7451) were also downloaded [25, 26, 27, 28, 29]. Additionally, two transcriptome‐level datasets (accession numbers: GSE84844 and GSE143153) were utilized for validation in this study [31, 37]. To assess model specificity, two datasets related to RA were included (accession numbers: GSE153015 and GSE224842). Details of the platform, number of HCs and patients, and data categories for each dataset are provided in Table 1 and Table S16.

### 5.2 Single‐cell RNA sequencing data analysis

#### 5.2.1 Data quantification, quality control, and integration

The scRNA‐seq data from previous SjS studies were reanalyzed. Specifically, Cell Ranger (version 7.0) from 10x Genomics (https://support.10xgenomics.com/single-cell-geneexpression/software/overview/welcome) was used to map and quantify the FASTQ data against the GRCh38 Human reference genome [10]. The “Seurat” (v4.3.0) R package was then applied for quality control, filtering out cells with detected genes fewer than 300 or greater than 7000, a total gene expression exceeding 100,000, mitochondrial content greater than 10%, and red blood cell content greater than 3% [38].

For normalization, Seurat's “NormalizeData()” function was used to normalize gene expression for each cell by scaling the data based on the total expression per cell. This step ensures gene expression levels are comparable across cells, reducing the influence of sequencing depth. Data were then scaled using Seurat's “ScaleData()” function, which centers each gene expression value by subtracting the mean and scaling by the standard deviation, preventing genes with larger variances from disproportionately affecting the analysis.

Next, Seurat's “Harmony” algorithm was used for data integration, correcting for batch effects between samples. Harmony aligns shared biological signals across datasets while removing technical variation, operating in the principal component analysis (PCA) space and adjusting principal components (PCs) to minimize batch‐related differences while preserving biological variance. After batch correction, dimensionality reduction was performed using PCA to project the data into a lower dimensional space. Subsequently, t‐distributed stochastic neighbor embedding (t‐SNE) and Uniform Manifold Approximation and Projection (UMAP) were applied for 2D visualization.

#### 5.2.2 Selection of a suitable resolution

The “clustree” R package was used to visualize cluster interactions across different resolutions (Figure S1A,B) and determine the optimal resolution for downstream analysis [11]. As shown in the figure, larger clusters (*e.g*., cluster 0) did not subdivide when the resolution exceeded 0.8, while smaller clusters (*e.g*., clusters 8 and 12) became increasingly intertwined, suggesting potential over‐clustering. Therefore, a resolution of 0.8 was selected for the preliminary grouping in subsequent analyses.

#### 5.2.3 Cell annotation from different techniques

For each cell subset identified through clustering, cell annotations were first performed using the “SingleR” package with the preloaded Human Primary Cell Atlas (HPCA) dataset (Figure S1C) [39]. The HPCA dataset, derived from the GEO repository, consists of 713 microarray samples classified into 38 major cell types [40]. The “SingleR” algorithm calculated the correlation between each cell in our dataset and the 713 reference samples, assigning the cell type label with the highest average correlation to each individual cell.

In addition, precise cell annotations were further refined using marker genes from the “CellMarker” database [12] and verified markers from the literature [22]. The “CellMarker” database includes 13,605 cell markers for 467 cell types across 158 human tissues and sub‐tissues. Sun et al. [22] classified CD4^+^ and CD8^+^ T cells into more specific subgroups based on their functional characteristics.

#### 5.2.4 Major cell type annotation: Density map

The “Nebulosa” R package [41], which utilizes kernel density estimation, was employed for advanced cell feature analysis. By integrating similarities between cells, Nebulosa enables the recovery of signals from gene deletions and facilitates the identification of double‐positive cell populations for specific genes. This function, in contrast to “Seurat,” provides enhanced visualization and analysis of cell populations based on gene expression patterns.

#### 5.2.5 Identification of differentially expressed genes

To identify specific highly expressed genes within each cluster, the “FindAllMarkers” function from Seurat was used. Genes showing a minimum log_2_‐fold change (logfc. threshold) > 0.25 in mean expression relative to all other clusters, and with adjusted *p*‐values < 0.05, were considered differentially expressed. *p*‐values were adjusted using Bonferroni correction. The top 10 most significantly expressed genes per cluster were selected for heatmap visualization (Figure S1D). Similarly, differential gene expression between SjS patients and HCs was analyzed using the “FindMarkers” function. Genes with a logfc. threshold > 0.25, pct.1 ‐ pct.2 > 0.1, and pct.2 < 0.5 were classified as highly expressed in each cluster. Differential gene expression was then displayed using a cross‐volcano map created with the “ggplot2” R package, omitting nonsignificant *p*‐values to emphasize the most biologically relevant findings.

There were two primary objectives for differential expression analysis in this study:

1) Defining Cell Subpopulations: The “FindAllMarkers” function was first used to identify genes that differentiate each cluster from others. These genes served as annotation markers to define cellular identities and subtypes (*e.g*., distinguishing subsets within γδ T cells). These markers are critical for consistent cell identification, but they are not necessarily disease‐specific, as their expression can vary across different physiological and pathological contexts.
2) Identifying Disease‐Associated Expression: The “FindMarkers” function was then used to compare gene expression within specific cell subpopulations between SjS patients and HCs. Genes identified in this step represent the disease‐associated molecular phenotype specific to each cell type in SjS.

#### 5.2.6 Single‐cell GSS

Single‐cell GSS is commonly employed in single‐cell RNA‐seq data analysis to capture cell‐type‐specific variability and heterogeneity by leveraging functional gene sets. In this study, single‐cell gene scoring was performed using the R package “AUCell” [42].

### 5.3 Gene ontology (GO) enrichment analysis

Functional (GO terms) analysis of the identified subgroups was conducted using the “compareCluster” function from the “clusterProfiler” R package [43], to determine significantly enriched molecular functions (MF), cellular components (CC), and biological processes (BP). Initially, gene IDs were converted from SYMBOL to ENTREZID using the “bitr“ function in the "org.Hs.eg.db”R package [44]. The subsequent functional analysis was applied to multiple gene sets simultaneously with the “compareCluster” function. GO terms with *p*‐values < 0.05 were considered significantly enriched for the respective gene sets (Figure 3F and Figures S3E and S4E). Network plots linking genes to GO terms are shown in Figure S6B,D,E.

### 5.4 Risk assessment model construction and evaluation

To minimize the risk of overfitting and information leakage, all feature selection procedures were strictly confined to the derivation cohort. Candidate genes were selected based on three criteria: (1) differentially expressed genes with higher expression in SjS patients within the four significantly altered clusters, (2) cluster‐specific signature genes, and (3) genes involved in pathways enriched in SjS. These procedures were exclusively applied to the training datasets.

Variables derived from the training cohort were then entered into a Least Absolute Shrinkage and Selection Operator (LASSO) regression model [45], using the “glmnet” R package (https://CRAN.R-project.org/package=glmnet). Model tuning was conducted via 10‐fold cross‐validation (cv.glmnet function) within the training data, and the optimal regularization parameter was determined by selecting the lambda.min value corresponding to the minimum mean squared error.

Six publicly available gene expression datasets (GSE208260, GSE159574, GSE127952, GSE97614, GSE40611, and GSE7451) were used for independent validation. These datasets, generated on different platforms (RNA‐seq and microarrays), were analyzed separately to avoid cross‐platform bias, ensuring that confounding factors from batch correction methods were not introduced. No additional feature selection was performed on the subsequent validation datasets.

For model evaluation, risk scores were calculated for each dataset using GSVA [46]. GSVA scores were computed sample‐wise based on the gene sets defined in the training cohort, maintaining the independence between training and validation datasets.

To assess the robustness and generalizability of the identified gene clusters, RF, an ensemble learning algorithm based on multiple decision trees, was employed as an additional validation approach. RF classifiers were trained for each dataset using the candidate gene set derived from the LASSO model to differentiate between SjS patients and HCs. Models were constructed with 500 trees (ntree = 500), and the number of variables sampled at each split was set to the square root of the total input gene count. A fixed random seed (set.seed(123)) ensured reproducibility. Variable importance was evaluated using the MDA metric, which measures the reduction in classification accuracy when a specific variable is permuted. Genes with an MDA value exceeding a predefined threshold were considered key contributors to disease classification. RF analyses were conducted independently across six datasets, and genes consistently identified as significant across datasets or cell populations were integrated to define a robust candidate gene set. Model performance was assessed using standard classification metrics and validated in independent external datasets.

### 5.5 Establishment of PPI network and identification of hub‐genes

The PPI network was constructed using the STRING database (http://string-db.org; version 11.5), from which all PPI data were downloaded [47]. The complete protein network data and protein annotation information were retrieved, and the SjS‐related protein interactions were extracted and visualized using Cytoscape 3.9.1. The MCODE clustering algorithm [48] was applied to identify densely connected regions in the PPI network, allowing for the extraction of highly interacting hub genes.

### 5.6 Verification of SjS.Sig

To evaluate the classification performance of SjS. Sig as a diagnostic tool for SjS, the R package “cancerclass” [49] was employed for validation and testing. The cancerclass protocol begins with feature selection, followed by nearest centroid classification and predictor construction. A ROC curve with an area under the curve (AUC) between 0.85 and 1.0 was considered excellent, indicating robust predictive performance [50]. The Wilson method, implemented in the cancerclass R package, calculated binomial confidence intervals for sensitivity and specificity. Fisher's exact test was applied to categorical variables, and all confidence intervals were reported as two‐sided binomial 95% confidence intervals.

### 5.7 RNA isolation and real‐time quantitative PCR (qPCR)

Peripheral blood was collected, diluted with an equal volume of PBS, and PBMCs were isolated using Ficoll‐Hypaque solution after differential centrifugation. RNA was extracted using a Total RNA Extraction Kit (AG21022, Accurate Biotechnology). cDNA was synthesized from 500 ng of total RNA using a Reverse Transcription Premix Kit (AG11706, Accurate Biotechnology). Real‐time quantitative PCR was conducted using a SYBR Green Premix qPCR Kit (AG11701, Accurate Biotechnology) on a Bio‐Rad CFX96 (USA). Primers for real‐time quantitative PCR are listed in Table S17.

### 5.8 Western blot analysis

Protein expression was assessed by Western blot. PBMCs from an additional eight participants were lysed, and protein concentrations were determined using a bicinchoninic acid assay assay (G2026, Servicebio). Equal protein amounts were separated by sodium dodecyl sulfate–polyacrylamide gel electrophoresis (PG112, Yeasen) and transferred to PVDF membranes (G605‐0.45, Servicebio). Membranes were incubated with primary antibodies against PSMB8 (14859‐1‐AP, Proteintech), HCLS1 (25003‐1‐AP, Proteintech), and GIMAP4 (11852‐1‐AP, Proteintech), followed by horseradish peroxidase‐conjugated secondary antibodies (SA00001‐2, Proteintech). Protein bands were visualized using an enhanced chemiluminescence substrate (P0018AS, Beyotime), and band intensities were quantified and normalized to β‐Actin (GB15003, Servicebio).

### 5.9 FACS analysis

Isolated PBMCs were resuspended in Cell Staining Buffer (Biolegend) and incubated on ice for 15 min to block nonspecific binding. The cells were then stained with fluorescent‐labeled antibodies at room temperature for 20 min. Following staining, the cells were analyzed using a BECKMAN COULTER CytoFLEX flow cytometer, with FlowJo software used for data analysis. The antibodies employed included allophycocyanin anti‐human CD8 (1 μL per 1 × 10^6^ cells, Biolegend, 344722), PE‐Cy7 Rat Anti‐Human CCR7 (CD197) (1 μL per 1 × 10^6^ cells, BD Pharmingen, 557648), fluorescein isothiocyanate anti‐human CD7 (1 μL per 1 × 10^6^ cells, Biolegend, 343104), Anti‐T cell receptor gamma delta (TCR‐γ/δ) phycoerythrin (PE) (1 μL per 1 × 10^6^ cells, BD, 333141), CD152 (CTLA‐4) monoclonal antibody (14D3), PE (1 μL per 1 × 10^6^ cells, Invitrogen, 12‐1529‐41), Alexa Fluor 647 anti‐mouse/rat/human *FOXP3* (1 μL per 1 × 10^6^ cells, Biolegend, 320013), and Pe‐Cyanine7 Anti‐Human CD25 (Bc96) (1 μL per 1 × 10^6^ cells, Tonbo, 60‐0259‐T100).

### 5.10 Statistical analysis

Statistical analysis was performed using R (version 4.1.3). For comparisons of flow cytometry and qPCR data, a two‐tailed Student's *t*‐test was applied. A *p*‐value < 0.05 was considered statistically significant. For multiple comparison analyses, the Benjamini–Hochberg (BH) procedure was applied to control the false discovery rate (FDR), adjusting *p*‐values to reduce the likelihood of false positives. Adjusted *p*‐values are reported where applicable.

## AUTHOR CONTRIBUTIONS

**Meili Gao**: Methodology; investigation. **Ling Yan**: Methodology; data curation, writing—review & editing; writing—original draft. **Shumin Cheng**: Methodology; software; data curation; investigation; validation; formal analysis; supervision; visualization; writing—review & editing; writing—original draft; conceptualization. **Yan Li**: Methodology; data curation; writing—review & editing; writing—original draft. **Yi Zhang**: Methodology; funding acquisition; investigation. **Huacheng Wang**: Data curation; investigation. **Jingyun Ma**: Data curation. **Ziheng Ye**: Methodology; data curation; software; writing—review & editing; writing—original draft. **Zhaofan Luo**: Data curation; investigation; supervision. **Yuhong Liang**: Methodology. **Yilin Wu**: Data curation; methodology. **Zhen Zhang**: Writing—original draft; writing—review & editing; data curation; methodology. **Chao Qin**: Methodology; investigation. **Ke Zhu**: Methodology. **Jun Liang**: Methodology; supervision; resources. **Kamran Ullah**: Methodology; supervision. **Yun Leng**: Methodology. **Yingqian Mo**: Methodology; supervision; funding acquisition; resources. **Guan Yang**: Methodology; supervision; resources. **Haiyan Yan**: Methodology, investigation. **Bihui Huang**: Funding acquisition; methodology; supervision; project administration; conceptualization; resources. All authors have read the final manuscript and approved it for publication.

## CONFLICT OF INTEREST STATEMENT

The authors declare no conflicts of interest.

## ETHICS STATEMENT

All participants provided written informed consent. This study was approved by the Medical Ethics Committees of Sun Yat‐sen Memorial Hospital (Approval No. SYSEC‐KY‐KS‐2018‐012) and Shenshan Medical Center, Sun Yat‐sen Memorial Hospital, Sun Yat‐sen University (Approval No. 2023‐SSKY‐575), and all procedures adhered to the Declaration of Helsinki. Written informed consent was obtained from all participants prior to their involvement.

## Supporting information


**Figure S1:** Re‐analysis of scRNA‐seq dataset ‐ GSE157278.
**Figure S2:** Cluster comparative analysis of the healthy control group and SjS patient group in GSE157278.
**Figure S3:** Correlation analysis of γδ T cell subpopulations in GSE157278.
**Figure S4:** Correlation analysis of *CD4* ^+^ T cell subsets in GSE157278.
**Figure S5:** Gene expression signatures that can characterize *TRDC* γδ T cells and *Helios FOXP3* ^lo^ *CD4* ^+^ Treg subsets.
**Figure S6:** LASSO convergence of Cluster 3 (Naïve *CD8* ^+^ T cell) across six GEO datasets.
**Figure S7:** LASSO convergence of Cluster 15 (*TRDC* γδ T cell) across six GEO datasets.
**Figure S8:** LASSO convergence of Cluster 17 (*Helios FOXP3* ^lo^ *CD4* ^+^ Treg) across six GEO datasets.
**Figure S9:** LASSO convergence of Cluster 19 (*CTLA*‐*4⁺ CD8* ^+^ Inhibitory) across six GEO datasets.
**Figure S10:** Analysis of 3 SjS‐specific cell subsets.
**Figure S11:** Validation of Random Forest model prediction performance in independent datasets.
**Figure S12:** Violin plots of the expression of four key cell subtypes associated with SjS DEGs in healthy and SjS patients.
**Figure S13:** Protein expression levels of PSMB8, HCLS1, and GIMAP4 are increased in SjS compared to non‐SjS samples.
**Figure S14:** Random Forest variable importance rankings for Naïve *CD8*⁺ T cells and *TRDC* γδ T cells across six GEO datasets.
**Figure S15:** Random Forest variable importance rankings for *Helios FOXP3* ^lo^ *CD4*⁺ Treg and *CTLA‐4*⁺ *CD8*⁺ inhibitory subsets across six GEO datasets.
**Figure S16:** Validation of prediction models across different datasets.
**Figure S17:** Comparison heatmap with GSE157278.

**Table S1:** The scRNA‐seq dataset ‐ GSE157278, the percentage of each cluster cell number in total cell number between the healthy controls (HCs) and patients with Sjögren's syndrome (SjS).
**Table S2:** Genes highly expressed in naïve *CD8* ^+^ T cells (avg_log_2_FC > 0.25).
**Table S3:** Differential expression analysis in naïve *CD8* ^+^ T cells.
**Table S4:** Pathway enrichment analysis in *CD8* ^+^ T cells.
**Table S5:** Genes highly expressed in *CTLA*‐*4⁺ CD8* ^+^ Inhibitory.
**Table S6:** Genes highly expressed in *TRDC* γδ T cells.
**Table S7:** Differential expression analysis in *TRDC* γδ T cell (cluster 15).
**Table S8:** Pathway enrichment analysis in γδ T cells.
**Table S9:** Genes highly expressed in *Helios FOXP3* ^lo^ *CD4* ^+^ Treg.
**Table S10:** Differential expression analysis in *Helios FOXP3* ^lo^ *CD4* ^+^ Treg (cluster 17).
**Table S11:** Pathway enrichment analysis in *CD4* ^+^ T cells.
**Table S12:** Feature gene sets of four SjS‐associated cell subpopulations.
**Table S13:** Clinical and laboratory features of patients with SjS and non‐SS control studied for FACS detection.
**Table S14:** Clinical and laboratory features of patients with Sj­S and non‐SS control studied for qPCR detection.
**Table S15:** Comparison of original and previous studies on markers and cell subsets.
**Table S16:** GEO datasets for rheumatoid arthritis analysis.
**Table S17:** Sequences of primers used in the PCR assay.

## Data Availability

All data analyzed in this study are publicly available. The single‐cell RNA sequencing (scRNA‐seq) data of Sjögren's syndrome (SjS) patients and healthy controls (HCs) were obtained from the Gene Expression Omnibus (GEO) under accession number GSE157278. The transcriptome‐level datasets used for model training and validation are available in GEO under the following accession numbers: GSE208260, GSE159574, GSE127952, GSE97614, GSE40611, GSE7451, GSE84844, and GSE143153. The datasets for model specificity assessment (rheumatoid arthritis, RA) are available under GEO accession numbers GSE153015 and GSE224842. The data and scripts used are saved in GitHub https://github.com/chengsm06-oss/Cheng2026iMetaOmics. Supplementary materials (figures, tables, graphical abstract, slides, videos, Chinese translated version and updated materials) may be found in the online DOI or iMetaOmics Science http://www.imeta.science/imetaomics.

## References

[1] Qin, Baodong , Jiaqi Wang , Zaixing Yang , Min Yang , Ning Ma , Fenglou Huang , and Renqian Zhong . 2015. “Epidemiology of Primary Sjögren's Syndrome: A Systematic Review and Meta‐Analysis.” Annals of the Rheumatic Diseases 74: 1983–1989. 10.1136/annrheumdis-2014-205375 24938285

[2] Psianou, Konstantia , Ioannis Panagoulias , Anastasios D. Papanastasiou , Anne‐Lise de Lastic , Maria Rodi , Panagiota I. Spantidea , Søren E. Degn , Panagiotis Georgiou , and Athanasia Mouzaki . 2018. “Clinical and Immunological Parameters of Sjögren's Syndrome.” Autoimmunity Reviews 17: 1053–1064. 10.1016/j.autrev.2018.05.005 30103041

[3] Nocturne, Gaëtane , and Xavier Mariette . 2013. “Advances in Understanding the Pathogenesis of Primary Sjögren's Syndrome.” Nature Reviews Rheumatology 9: 544–556. 10.1038/nrrheum.2013.110 23857130

[4] Thorlacius, Gudny Ella , Albin Björk , and Marie Wahren‐Herlenius . 2023. “Genetics and Epigenetics of Primary Sjögren Syndrome: Implications for Future Therapies.” Nature Reviews Rheumatology 19: 288–306. 10.1038/s41584-023-00932-6 PMC10010657 36914790

[5] Cruz‐Tapias, Paola , Adriana Rojas‐Villarraga , Shannon Maier‐Moore , and Juan‐Manuel Anaya . 2012. “HLA and Sjögren's Syndrome Susceptibility. A Meta‐Analysis of Worldwide Studies.” Autoimmunity Reviews 11: 281–287. 10.1016/j.autrev.2011.10.002 22001416

[6] Li, Yongzhe , Kunlin Zhang , Hua Chen , Fei Sun , Juanjuan Xu , Ziyan Wu , Ping Li , et al. 2013. “A Genome‐Wide Association Study in Han Chinese Identifies a Susceptibility Locus for Primary Sjögren's Syndrome at 7q11. 23.” Nature Genetics 45: 1361–1365. 10.1038/ng.2779 24097066

[7] Lessard, Christopher J. , He Li , Indra Adrianto , John A. Ice , Astrid Rasmussen , Kiely M. Grundahl , Jennifer A. Kelly , et al. 2013. “Variants at Multiple Loci Implicated in Both Innate and Adaptive Immune Responses Are Associated With Sjögren's Syndrome.” Nature Genetics 45: 1284–1292. 10.1038/ng.2792 PMC3867192 24097067

[8] Zhang, Shanshan , Jiaan Zhu , Xia Zhang , Jing He , and Jianguo Li . 2016. “Assessment of the Stiffness of Major Salivary Glands in Primary Sjögren's Syndrome through Quantitative Acoustic Radiation Force Impulse Imaging.” Ultrasound in Medicine & Biology 42: 645–653. 10.1016/j.ultrasmedbio.2015.11.009 26715188

[9] Tashbayev, Behzod , Shermin Rusthen , Alix Young , Bente Brokstad Herlofson , Lene Hystad Hove , Preet Bano Singh , Morten Rykke , et al. 2017. “Interdisciplinary, Comprehensive Oral and Ocular Evaluation of Patients with Primary Sjögren's Syndrome.” Scientific Reports 7: 10761. 10.1038/s41598-017-10809-w PMC5589846 28883442

[10] Hong, Xiaoping , Shuhui Meng , Donge Tang , Tingting Wang , Liping Ding , Haiyan Yu , Heng Li , et al. 2021. “Single‐Cell RNA Sequencing Reveals the Expansion of Cytotoxic CD4+ T Lymphocytes and a Landscape of Immune Cells in Primary Sjögren's Syndrome.” Frontiers in Immunology 11: 594658. 10.3389/fimmu.2020.594658 PMC7884617 33603736

[11] Zappia, Luke , and Alicia Oshlack . 2018. “Clustering Trees: A Visualization for Evaluating Clusterings at Multiple Resolutions.” Gigascience 7: giy083. 10.1093/gigascience/giy083 PMC6057528 30010766

[12] Zhang, Xinxin , Yujia Lan , Jinyuan Xu , Fei Quan , Erjie Zhao , Chunyu Deng , Tao Luo , et al. 2019. “CellMarker: A Manually Curated Resource of Cell Markers in Human and Mouse.” Nucleic Acids Research 47: D721–D728. 10.1093/nar/gky900 PMC6323899 30289549

[13] Choi, Sung Min , Hi Jung Park , Eun A. Choi , Kyeong Cheon Jung , and Jae Il Lee . 2021. “Cellular Heterogeneity of Circulating CD4+ CD8+ Double‐Positive T Cells Characterized by Single‐Cell RNA Sequencing.” Scientific Reports 11: 23607. 10.1038/s41598-021-03013-4 PMC8655006 34880348

[14] Lu, Huanzi , Wenxiao Dai , Junyi Guo , Dikan Wang , Shuqiong Wen , Lisa Yang , Dongjia Lin , et al. 2020. “High Abundance of Intratumoral γδ T Cells Favors a Better Prognosis in Head and Neck Squamous Cell Carcinoma: A Bioinformatic Analysis.” Frontiers in Immunology 11: 573920. 10.3389/fimmu.2020.573920 PMC7555127 33101298

[15] Morgan, Dhakshayini , and Vinay Tergaonkar . 2022. “Unraveling B Cell Trajectories at Single Cell Resolution.” Trends in Immunology 43: 210–229. 10.1016/j.it.2022.01.003 35090788

[16] Szabo, Peter A. , Hanna Mendes Levitin , Michelle Miron , Mark E. Snyder , Takashi Senda , Jinzhou Yuan , Yim Ling Cheng , et al. 2019. “Single‐Cell Transcriptomics of Human T Cells Reveals Tissue and Activation Signatures in Health and Disease.” Nature Communications 10: 4706. 10.1038/s41467-019-12464-3 PMC6797728 31624246

[17] Tang‐Huau, Tsing‐Lee , Paul Gueguen , Christel Goudot , Mélanie Durand , Mylène Bohec , Sylvain Baulande , Benoit Pasquier , Sebastian Amigorena , and Elodie Segura . 2018. “Human in Vivo‐Generated Monocyte‐Derived Dendritic Cells and Macrophages Cross‐Present Antigens Through a Vacuolar Pathway.” Nature Communications 9: 2570. 10.1038/s41467-018-04985-0 PMC6028641 29967419

[18] Tremble, Liam Friel , Mark McCabe , Sidney P. Walker , Siobhán McCarthy , Réiltín F Tynan , Suzanne Beecher , Réiltín Werner , et al. 2020. “Differential Association of CD68+ and CD163+ Macrophages with Macrophage Enzymes, Whole Tumour Gene Expression and Overall Survival in Advanced Melanoma.” British Journal of Cancer 123: 1553–1561. 10.1038/s41416-020-01037-7 PMC7653046 32843682

[19] Villani, Alexandra‐Chloé , Rahul Satija , Gary Reynolds , Siranush Sarkizova , Karthik Shekhar , James Fletcher , Morgane Griesbeck , et al. 2017. “Single‐Cell RNA‐seq Reveals New Types of Human Blood Dendritic Cells, Monocytes, and Progenitors.” Science 356: eaah4573. 10.1126/science.aah4573 PMC5775029 28428369

[20] Yang, Chao , Jason R. Siebert , Robert Burns , Zachary J. Gerbec , Benedetta Bonacci , Amy Rymaszewski , Mary Rau , et al. 2019. “Heterogeneity of Human Bone Marrow and Blood Natural Killer Cells Defined by Single‐Cell Transcriptome.” Nature Communications 10: 3931. 10.1038/s41467-019-11947-7 PMC6718415 31477722

[21] Yeung, Anthony K. , Carlos Villacorta‐Martin , Stephanie Hon , Jason R. Rock , and George J. Murphy . 2020. “Lung Megakaryocytes Display Distinct Transcriptional and Phenotypic Properties.” Blood Advances 4: 6204–6217. 10.1182/bloodadvances.2020002843 PMC7757004 33351116

[22] Sun, Yunfan , Liang Wu , Y. U. Zhong , Kaiqian Zhou , Yong Hou , Zifei Wang , Zefan Zhang , et al. 2021. “Single‐Cell Landscape of the Ecosystem in Early‐Relapse Hepatocellular Carcinoma.” Cell 184: 404–421.e16. 10.1016/j.cell.2020.11.041 33357445

[23] Pedicord, Virginia A. , Welby Montalvo , Ingrid M. Leiner , and James P. Allison . 2011. “Single Dose of anti–CTLA‐4 Enhances CD8+ T‐Cell Memory Formation, Function, and Maintenance.” Proceedings of the National Academy of Sciences 108: 266–271. 10.1073/pnas.1016791108 PMC3017182 21173239

[24] Voulgarelis, Michael , and Athanasios G. Tzioufas . 2010. “Pathogenetic Mechanisms in the Initiation and Perpetuation of Sjögren's Syndrome.” Nature Reviews Rheumatology 6: 529–537. 10.1038/nrrheum.2010.118 20683439

[25] Hu, Shen , Jianghua Wang , Jiska Meijer , Sonya Ieong , Yongming Xie , Tianwei Yu , et al. 2007. “Salivary Proteomic and Genomic Biomarkers for Primary Sjögren's Syndrome.” Arthritis & Rheumatism: Official Journal of the American College of Rheumatology 56: 3588–3600. 10.1002/art.22954 PMC2856841 17968930

[26] Horvath, Steve , Abu N. M. Nazmul‐Hossain , Rodney P. E. Pollard , Frans G. M. Kroese , Arjan Vissink , Cees G. M. Kallenberg , et al. 2012. “Systems Analysis of Primary Sjögren's Syndrome Pathogenesis in Salivary Glands Identifies Shared Pathways in Human and a Mouse Model.” Arthritis Research & Therapy 14: R238. 10.1186/ar4081 PMC3674589 23116360

[27] Luo, Jing , Xin Liao , Lihe Zhang , Xin Xu , Senhong Ying , Mengjiao Yu , Lixia Zhu , Suxian Lin , and Xiaobing Wang . 2020. “Transcriptome Sequencing Reveals Potential Roles of ICOS in Primary Sjögren's Syndrome.” Frontiers in Cell and Developmental Biology 8: 592490. 10.3389/fcell.2020.592490 PMC7747463 33344450

[28] Vakrakou, Aigli G. , Alexandros Polyzos , Efstathia K. Kapsogeorgou , Dimitris Thanos , and Menelaos N. Manoussakis . 2018. “Perturbation of Transcriptome in Non‐Neoplastic Salivary Gland Epithelial Cell Lines Derived from Patients with Primary Sjögren's Syndrome.” Data in Brief 17: 194–199. 10.1016/j.dib.2017.12.023 PMC5988450 29876386

[29] Verstappen, Gwenny M. , Lu Gao , Sarah Pringle , Erlin A. Haacke , Bert van der Vegt , Silvia C. Liefers , Vishal Patel , et al. 2021. “The Transcriptome of Paired Major and Minor Salivary Gland Tissue in Patients with Primary Sjögren's Syndrome.” Frontiers in Immunology 12: 681941. 10.3389/fimmu.2021.681941 PMC8291032 34295332

[30] Chan, Derrick V. , Heather M. Gibson , Barabara M. Aufiero , Adam J. Wilson , Mikehl S. Hafner , Qing‐Sheng Mi , et al. 2014. “Differential CTLA‐4 Expression in Human CD4+ Versus CD8+ T Cells Is Associated with Increased NFAT1 and Inhibition of CD4+ Proliferation.” Genes & Immunity 15: 25–32. 10.1038/gene.2013.57 PMC4284071 24173147

[31] Tasaki, Shinya , Katsuya Suzuki , Ayumi Nishikawa , Yoshiaki Kassai , Maiko Takiguchi , Rina Kurisu , Yuumi Okuzono , et al. 2017. “Multiomic Disease Signatures Converge to Cytotoxic CD8 T Cells in Primary Sjögren's Syndrome.” Annals of the Rheumatic Diseases 76: 1458–1466. 10.1136/annrheumdis-2016-210788 PMC5738597 28522454

[32] Heinonen, Mirkka T. , Antti‐Pekka Laine , Cilla Söderhäll , Olena Gruzieva , Sini Rautio , Erik Melén , Göran Pershagen , et al. 2015. “GIMAP GTPase Family Genes: Potential Modifiers in Autoimmune Diabetes, Asthma, and Allergy.” The Journal of Immunology 194: 5885–5894. 10.4049/jimmunol.1500016 PMC4456634 25964488

[33] Newby, Brittney N. , and Clayton E. Mathews . 2017. “Type I Interferon Is a Catastrophic Feature of the Diabetic Islet Microenvironment.” Frontiers in Endocrinology 8: 232. 10.3389/fendo.2017.00232 PMC5604085 28959234

[34] Yao, Yuan , Jin‐Fen Ma , Christopher Chang , Ting Xu , Cai‐Yue Gao , M Eric Gershwin , et al. 2021. “Immunobiology of T Cells in Sjögren's Syndrome.” Clinical Reviews in Allergy & Immunology 60: 111–131. 10.1007/s12016-020-08793-7 32390096

[35] Nocturne, Gaetane , Jessica Tarn , Saida Boudaoud , James Locke , Corinne Miceli‐Richard , Eric Hachulla , Jean‐Jacques Dubost , et al. 2016. “Germline Variation of TNFAIP3 in Primary Sjögren's Syndrome‐Associated Lymphoma.” Annals of the Rheumatic Diseases 75: 780–783. 10.1136/annrheumdis-2015-207731 26338037

[36] Cinoku, Ilir I. , Kleio‐Maria Verrou , Evangelia Piperi , Michael Voulgarelis , Haralampos M. Moutsopoulos , and Clio P. Mavragani . 2021. “Interferon (IFN)‐stimulated Gene 15: A Novel Biomarker for Lymphoma Development in Sjögren's Syndrome.” Journal of Autoimmunity 123: 102704. 10.1016/j.jaut.2021.102704 34298409

[37] Joachims, Michelle L. , Kerry M. Leehan , Mikhail G. Dozmorov , Constantin Georgescu , Zijian Pan , Christina Lawrence , M. Caleb Marlin , et al. 2020. “Sjögren's Syndrome Minor Salivary Gland CD4+ Memory T Cells Associate with Glandular Disease Features and Have a Germinal Center T Follicular Helper Transcriptional Profile.” Journal of Clinical Medicine 9: 2164. 10.3390/jcm9072164 PMC7408878 32650575

[38] Hao, Yuhan , Stephanie Hao , Erica Andersen‐Nissen , William M. Mauck , Shiwei Zheng , Andrew Butler , Maddie J. Lee , et al. 2021. “Integrated Analysis of Multimodal Single‐Cell Data.” Cell 184: 3573–3587.e29. 10.1016/j.cell.2021.04.048 PMC8238499 34062119

[39] Aran, Dvir , Agnieszka P. Looney , Leqian Liu , Esther Wu , Valerie Fong , Austin Hsu , Suzanna Chak , et al. 2019. “Reference‐Based Analysis of Lung Single‐Cell Sequencing Reveals a Transitional Profibrotic Macrophage.” Nature Immunology 20: 163–172. 10.1038/s41590-018-0276-y PMC6340744 30643263

[40] Mabbott, Neil A. , J. Baillie , Helen Brown , Tom C. Freeman , and David A. Hume . 2013. “An Expression Atlas of Human Primary Cells: Inference of Gene Function from Coexpression Networks.” BMC Genomics 14: 632. 10.1186/1471-2164-14-632 PMC3849585 24053356

[41] Alquicira‐Hernandez, Jose , and Joseph E. Powell . 2021. “Nebulosa Recovers Single‐Cell Gene Expression Signals By Kernel Density Estimation.” Bioinformatics 37: 2485–2487. 10.1093/bioinformatics/btab003 33459785

[42] Aibar, Sara , Carmen Bravo González‐Blas , Thomas Moerman , Vân Anh Huynh‐Thu , Hana Imrichova , Gert Hulselmans , Florian Rambow , et al. 2017. “SCENIC: Single‐Cell Regulatory Network Inference and Clustering.” Nature Methods 14: 1083–1086. 10.1038/nmeth.4463 PMC5937676 28991892

[43] Wu, Tianzhi , Erqiang Hu , Shuangbin Xu , Meijun Chen , Pingfan Guo , Zehan Dai , Tingze Feng , et al. 2021. “Clusterprofiler 4.0: A Universal Enrichment Tool for Interpreting Omics Data.” The Innovation 2: 100141. 10.1016/j.xinn.2021.100141 PMC8454663 34557778

[44] Carlson, Marc , S. Falcon , H. Pages , and N. Li . 2019. Org. Hs. Eg. Db: Genome Wide Annotation for Human. 10.18129/B9.bioc.org.Hs.eg.db

[45] Tibshirani, Robert . 1996. “Regression Shrinkage and Selection Via the Lasso.” Journal of the Royal Statistical Society Series B: Statistical Methodology 58: 267–288. 10.1111/j.2517-6161.1996.tb02080.x

[46] Hänzelmann, Sonja , Robert Castelo , and Justin Guinney . 2013. “GSVA: Gene Set Variation Analysis for Microarray and RNA‐seq Data.” BMC Bioinformatics 14: 7. 10.1186/1471-2105-14-7 PMC3618321 23323831

[47] Szklarczyk, Damian , John H. Morris , Helen Cook , Michael Kuhn , Stefan Wyder , Milan Simonovic , Alberto Santos , et al. 2016. “The STRING Database in 2017: Quality‐Controlled Protein–Protein Association Networks, Made Broadly Accessible.” Nucleic Acids Research 45: D362–D368. 10.1093/nar/gkw937 PMC5210637 27924014

[48] Bader, Gary D. , and Christopher W. V. Hogue . 2003. “An Automated Method for Finding Molecular Complexes in Large Protein Interaction Networks.” BMC Bioinformatics 4: 2. 10.1186/1471-2105-4-2 PMC149346 12525261

[49] Jan, Budczies , Daniel Kosztyla , Christian von Törne , Albrecht Stenzinger , Silvia Darb‐Esfahani , Manfred Dietel , et al. 2014. “Cancerclass: An R Package for Development and Validation of Diagnostic Tests From High‐Dimensional Molecular Data.” Journal of Statistical Software 59: 1–19.

[50] Fluss, Ronen , David Faraggi , and Benjamin Reiser . 2005. “Estimation of the Youden Index and Its Associated Cutoff Point.” Biometrical Journal 47: 458–472. 10.1002/bimj.200410135 16161804

