# Supplementary material for: Gene expression signatures from single‐cell transcriptomics predict Sjögren's syndrome
Source: IMetaOmics. 2026 May 28;3(3):e70106. doi: 10.1002/imo2.70106 (PMC13674423; doi:10.1002/imo2.70106)
Supplement: Supplementary file 1 [file IMO2-3-e70106-s001.docx]

Supporting Information to

Gene expression signatures from single-cell transcriptomics predict Sjögren’s syndrome

**Running title**: Single-cell transcriptomic signatures predict SjS

Shumin Cheng^1#^, Ling Yan^1#^, Yilin Wu^2#^, Yan Li^1#^, Ziheng Ye^1#^, Zhen Zhang^3#^, Yi Zhang^1, 3^, Jingyun Ma^1^, Yuhong Liang^4^, Zhaofan Luo^1^, Huacheng Wang^1^, Meili Gao^5^, Chao Qin^1^, Ke Zhu^1, 3^, Yun Leng^1^, Kamran Ullah^6^, Jun Liang^1*^, Haiyan Yan^3*^, Guan Yang^7*^, Yingqian Mo^3, 8*^, Bihui Huang ^3, 9*^

^1^ The Seventh Affiliated Hospital, Sun Yat-sen University, Shenzhen 518107, China

^2^ Shenzhen University of Advanced Technology General Hospital, Shenzhen 518107, China

^3^ Shenshan Medical Center, Sun Yat-sen Memorial Hospital, Sun Yat-Sen University, Shanwei 516621, China

^4^ School of Pharmacy, Macau University of Science and Technology, Macau 990078, China

^5^ The Second People’s Hospital of Futian District, Shenzhen 518000, China

^6^ Department of Biology, The University of Haripur, Haripur KP, Pakistan

^7^ Department of Infectious Diseases and Public Health, City University of Hong Kong, Hong Kong SAR 999077, China.

^8^ Sun Yat-sen Memorial Hospital, Sun Yat-sen University, Guangzhou 510235, China

^9^ The People’s Hospital of Guangxi Zhuang Autonomous Region, Nanning 530021, China

^#^ These authors contributed equally: Shuming Cheng, Ling Yan, Yilin Wu, Yan Li, Ziheng Ye, and Zhen Zhang

* Correspondence: [huangbh7@mail.sysu.edu.cn](mailto:huangbh7@mail.sysu.edu.cn) (Bihui Huang), [moyingq@mail.sysu.edu.cn](mailto:moyingq@mail.sysu.edu.cn) (Yingqian Mo), [gyang25@cityu.edu.hk](mailto:gyang25@cityu.edu.hk) (Guan Yang), [yanhaiy@mail.sysu.edu.cn](mailto:yanhaiy@mail.sysu.edu.cn) (Haiyan Yan), and [liangjun@mail.sysu.edu.cn](mailto:liangjun@mail.sysu.edu.cn) (Jun Liang).

**Figure S1 Re-analysis of scRNA-seq dataset - GSE157278.** (A) Clustree at different clustering resolutions. Each row represents a resolution, the circle size represents the number of cells in the cluster, and the arrow represents the trajectory of the cell. (B) UMAP diagrams at different cluster resolutions. Different colors represent the 27 clusters obtained when the resolution is set to 1.2, the circle size represents the number of cells in the cluster, and the arrow represents the trajectory of the cells. (C) t-SNE plot after singleR active annotation. (D) DoHeatmap. Resolution selection 0.8, each cluster selects the top 5 specifically highly expressed genes.

**Figure S2 Cluster comparative analysis of the healthy control group and SjS patient group in GSE157278.** (A) t-SNE plot after clustering of the healthy control group. (B) t-SNE plot after clustering of the SjS patient group. (C) Boxplot of the percentage change of cell clusters between control and SjS groups. The *p*-value is calculated using a two-sided Wilcoxon test. The boxplot with a central horizontal line shows the median. Boxes span from the 25^th^ to the 75^th^ percentile, while points outside the whiskers indicate outliers.

**Figure S3 Correlation analysis of γδ T cell subpopulations in GSE157278.** (A) 3D pie chart of the proportion of each subpopulation of γδ T cells. (B) Violin plot of log-transformed expression of specifically expressed genes, showing statistically significant upregulation in each subpopulation of γδ T cells. (C) Bubble plot of highly expressed genes (TRGC2^hi^ γδ T cell, TRGC2^me^ γδ T cell, and TRDC γδ T cell) in each subgroup of γδ T cells. (D) Cross-volcano map of gene expression in TRDC γδ T cell subsets. (E) GO enrichment analysis map in each subpopulation of γδ T cells.

 **Figure S4 Correlation analysis of CD4^+^ T cell subsets in GSE157278.** (A) 3D pie chart of the proportion of each subset of CD4^+^ T cells. (B) Violin plot of log-transformed expression of specifically expressed genes, showing statistically significant upregulation in each CD4^+^ T cell subset. (C) Bubble plot of highly expressed genes (Naïve CD4^+^ T cell, CD25^+^ Foxp3^hi^ CD4^+^ Treg, Foxp3^hi^ CD4^+^ Treg, and Helios Foxp3^lo^ CD4^+^ Treg) in each subset of CD4^+^ T cells. (D) Gene expression cross volcano plot of Helios Foxp3^lo^ CD4^+^ Treg subpopulation. (E) GO enrichment analysis chart in each subpopulation of CD4^+^ T cells.

­ **Figure S5 Gene expression signatures that can characterize TRDC γδ T cells and Helios Foxp3^-^ CD4^+^ Treg subsets.** (A) AUCell score of the characteristic expression gene set of TRDC γδ T cell subpopulation, the violin plot shows that this gene set is more active in TRDC γδ T cells compared with other γδ T cell subpopulations. (B) AUCell scores for the signature expressed gene sets of the Helios Foxp3^lo^ CD4^+^ Treg subpopulation. (C) GSVA analysis of the characteristic expression gene sets (*n* = 23) related to SjS in the TRDC γδ T cell subpopulation in the 6 GEO test datasets after lasso analysis. (D) GSVA analysis of the characteristic expression gene sets (*n* = 19) associated with SjS in the Helios Foxp3^lo^ CD4^+^ Treg subpopulation in the six GEO test datasets after lasso regression analysis. Significant differences are indicated by **p* < 0.05, ***p* < 0.01, ****p* < 0.001, *****p* < 0.0001, ns: not significant, as shown in the corresponding panels.

**Figure S6 LASSO convergence of Cluster 3 (Naïve CD8^+^ T cell) across 6 GEO Datasets.**

**Figure S7 LASSO convergence of Cluster 15 (TRDC γδ T cell) across 6 GEO Datasets.**

**Figure S8 LASSO convergence of Cluster 17 (Helios Foxp3^lo^ CD4^+^ Treg) across 6 GEO Datasets.**

**Figure S9 LASSO convergence of Cluster 19 (CTLA-4 CD8^+^ Inhibitory) across 6 GEO Datasets.**

 **Figure S10 Analysis of 3 SjS-specific cell subsets.** (A) Heatmap of highly expressed genes in CD8^+^ T cells. (B) Network correlation map of GO terms enrichment in each subgroup of CD8^+^ T cells. (C) Heatmap of highly expressed genes in γδ T cells. (D) Network correlation map of GO terms of enrichment in each subgroup of γδ T cells. (E) Heatmap of highly expressed genes in CD4^+^ T cells. (F) Network association map of GO term enrichment in each subgroup of CD4^+^ T cells.

**Figure S11 Validation of prediction models across different datasets.** (A and B) Calibration plots showing the observed event rates against the mean predicted probabilities for SjS datasets GSE84844 (A) and GSE143153 (B). (C−F) ROC curves for rheumatoid arthritis datasets GSE153015 (C) and GSE224842 (E). Calibration plots for the rheumatoid arthritis datasets GSE153015 (D) and GSE224842 (F), showing observed event rates versus mean predicted probabilities.

**Figure S12 Violin plots of the expression of four key cell subtypes associated with SjS DEGs in healthy and SjS patients.** (A) Expression of DEGs in Cluster 3 (Naïve CD8^+^ T cells). (B) Expression of DEGs in Cluster 15 (γδ T cells). (C) Expression of DEGs in Cluster 17 (Helios Foxp3^lo^ CD4^+^ Treg) subgroup. (D) Expression of DEGs in Cluster 19 (CTLA-4 CD8^+^ Inhibitory) subgroup.

 **Figure S13 Protein expression levels of PSMB8, HCLS1, and GIMAP4 are increased in SjS compared to non-SjS samples.** (A) Western blot analysis showing the protein expression of PSMB8, HCLS1, and GIMAP4 in non-SjS and SjS samples. β-actin served as the loading control. (B−D) Quantification of relative protein expression levels of PSMB8 (B), HCLS1 (C), and GIMAP4 (D) in non-SjS (blue) and SjS (red) samples. The relative expression was normalized to β-actin levels. Significant differences between non-SjS and SjS are indicated by **p* < 0.05.

 **Figure S14 Random Forest variable importance rankings for Naïve CD8⁺ T cells and TRDC γδ T cells across six GEO datasets.** (A−F) Ranking of the most important variables identified by RF analysis in six GEO datasets (GSE127952, GSE159574, GSE202860, GSE86714, GSE7451, GSE40611) for Naïve CD8^+^ T cell subsets. These variables were ranked based on their importance in distinguishing Naïve CD8^+^ T cells across the different datasets. (G−L) Ranking of the most important variables identified by RF analysis in six GEO datasets (GSE127952, GSE159574, GSE202860, GSE86714, GSE7451, GSE40611) for TRDC γδ T cell subsets. These variables were ranked based on their importance in distinguishing TRDC γδ T cells across the different datasets.

 **Figure S15 Random Forest variable importance rankings for Helios Foxp3^lo^ CD4⁺ Treg and CTLA-4⁺ CD8⁺ inhibitory subsets across six GEO datasets.** (A−F) Ranking of the most important variables identified by RF analysis in six GEO datasets (GSE127952, GSE159574, GSE202860, GSE86714, GSE7451, GSE40611) for Helios Foxp3^lo^ CD4^+^ Treg subsets. These variables were ranked based on their importance in distinguishing Helios Foxp3^lo^ CD4^+^ Treg across the different datasets. (G–L) Ranking of the most important variables identified by RF analysis in six GEO datasets (GSE127952, GSE159574, GSE202860, GSE86714, GSE7451, GSE40611) for CTLA-4 CD8^+^ inhibitory subsets. These variables were ranked based on their importance in distinguishing CTLA-4 CD8^+^ inhibitory across the different datasets.

 **Figure S16 Validation of Random Forest model prediction performance in independent datasets.** The prediction performance of GSE143153 (A) and GSE84844 (B) was validated on the RF dataset.

**Figure S17 Comparison heatmap with GSE157278.** (A) Comparative heat map of CD4^+^ T cells. The top is the four types of CD4^+^ T cell subpopulations annotated in this study, the left is the marker gene, and the right is the annotated subpopulation in the original GSE157278. The bolded part is significant in SjS. (B) Comparative heat map of CD8^+^ T cells. The top is the four types of CD8^+^ T cell subpopulations annotated in this study, the left is the marker gene, and the right is the annotated subpopulation in the original GSE157278.
